# Supplementary material for: Supramolecular double-stranded Archimedean spirals and concentric toroids
Source: Nat Commun. 2020 Jul 17;11:3578. doi: 10.1038/s41467-020-17356-5 (PMC7368029; doi:10.1038/s41467-020-17356-5)
Supplement: Supplementary file 1 — Supplementary Information [file 41467_2020_17356_MOESM1_ESM.pdf]

Supramolecular double-stranded Archimedean spirals and concentric toroids

Norihiko Sasaki[1,2], Mathijs F. J. Mabesoone[3], Jun Kikkawa[2], Tomoya Fukui[2], Nobutaka Shioya[4], Takafumi Shimoaka[4], Takeshi Hasegawa[4], Hideaki Takagi[5], Rie Haruki[5], Nobutaka Shimizu[5], Shin-ichi Adachi[5], E. W. Meijer[3], Masayuki Takeuchi[2,*], Kazunori Sugiyasu[1,2,*]

1. Department of Materials Physics and Chemistry, Graduate School of Engineering, Kyushu University, 744 Moto-oka, Nishi-ku, Fukuoka 819-0395, Japan
2. National Institute for Materials Science, 1-2-1 Sengen, Tsukuba, Ibaraki 305-0047, Japan
3. Laboratory of Macromolecular and Organic Chemistry and the Institute for Complex Molecular Systems, Eindhoven University of Technology, P.O. Box 513, Eindhoven 5600 MB, The Netherlands
4. Laboratory of Chemistry for Functionalized Surfaces, Division of Environmental Chemistry, Institute for Chemical Research, Kyoto University, Gokasho, Uji, Kyoto 611-0011, Japan
5. Photon Factory, Institute of Materials Structure Science, High Energy Accelerator Research Organization, Tsukuba, Ibaraki 305-0801, Japan

E mail:
TAKEUCHI.Masayuki@nims.go.jp
SUGIYASU.Kazunori@nims.go.jp

Table of Contents

1. Materials and Methods 2
2. Synthesis and Characterization 3
3. Supplementary Figures and Tables 14
4. Supplementary References 40

1. Materials and Methods

Unless otherwise noted, reagents and solvents were purchased from commercial suppliers without further purification. Air- and/or water-sensitive reactions were conducted under argon atmosphere using dry solvents. Compound **6HH**[1], **S5**[2] and **S6**[3] were prepared according to reported procedures.

Nuclear magnetic resonance (NMR) spectra were recorded on a JEOL ECS-400 (400 MHz) spectrometer. All chemical shifts are reported in parts per million (ppm) from tetramethylsilane (0 ppm for ^{1}H), residual $CHCl_3$ (77 ppm for ^{13}C), or hexafluorobenzene (−162 ppm for ^{19}F) as an internal standard. Matrix-assisted laser desorption ionization time-of-flight (MALDI-TOF) mass spectra were obtained using a Shimadzu Axima-CFR plus station. Melting points were determined with a Yanako NP-500P micro melting point apparatus. Ultraviolet–visible absorption spectra were recorded using a quartz cuvette of 10, 2, or 1 mm path length on a Jasco V-630 spectrophotometer equipped with a Jasco ETCS-761 cell holder for temperature control or Jasco FS-110 spectrophotometer equipped with a Jasco ETCS-761 cell holder for temperature control. Fourier transform infrared (FT-IR) spectroscopic analyses were performed on a Jasco FT/IR-4700. Spin-coating was performed using an Oshigane SC-300. Atomic force microscopy (AFM) was performed on a Bruker model MultiMode 8 atomic force microscope under ambient conditions in the scan assist mode. Silicon cantilevers (SCANASYST-AIR) with a spring constat of 0.4 N/m and a frequency of 70 kHz were used. AFM images were analyzed with Bruker Nanoanalysis and ImageJ. The molecular modeling was carried out with the Spartan ’14 package (Wavefunction, Inc., Irvine. CA).

Small angle X-ray scattering (SAXS)

SAXS measurement was conducted at the BL-15A2 of the Photon Factory of the High Energy Accelerator Research Organization (KEK) in Tsukuba, Japan[4]. PILATUS3 2M (DECTRIS) was used as a detector. The X-ray wavelength was 1.213 Å and a sample detector distance was 1090 mm. The temperature was maintained at around 293 K (room temperature). The 2D scattering data were radially averaged to yield 1D scattering intensity data. These data were then normalized using pure water as a reference, and the following subtraction of the background (quartz glass window and solvent) gave absolute scattering intensity I(q) in cm^{-1}. All data reduction was carried out using software package SAngler[5]. The magnitude of the scattering vector is given by $q = (4\pi/\lambda)\sin\theta/2$, where λ is the X-ray wavelength a d is the scattering angle.

2. Synthesis and Characterization

Synthesis of compound 6FF

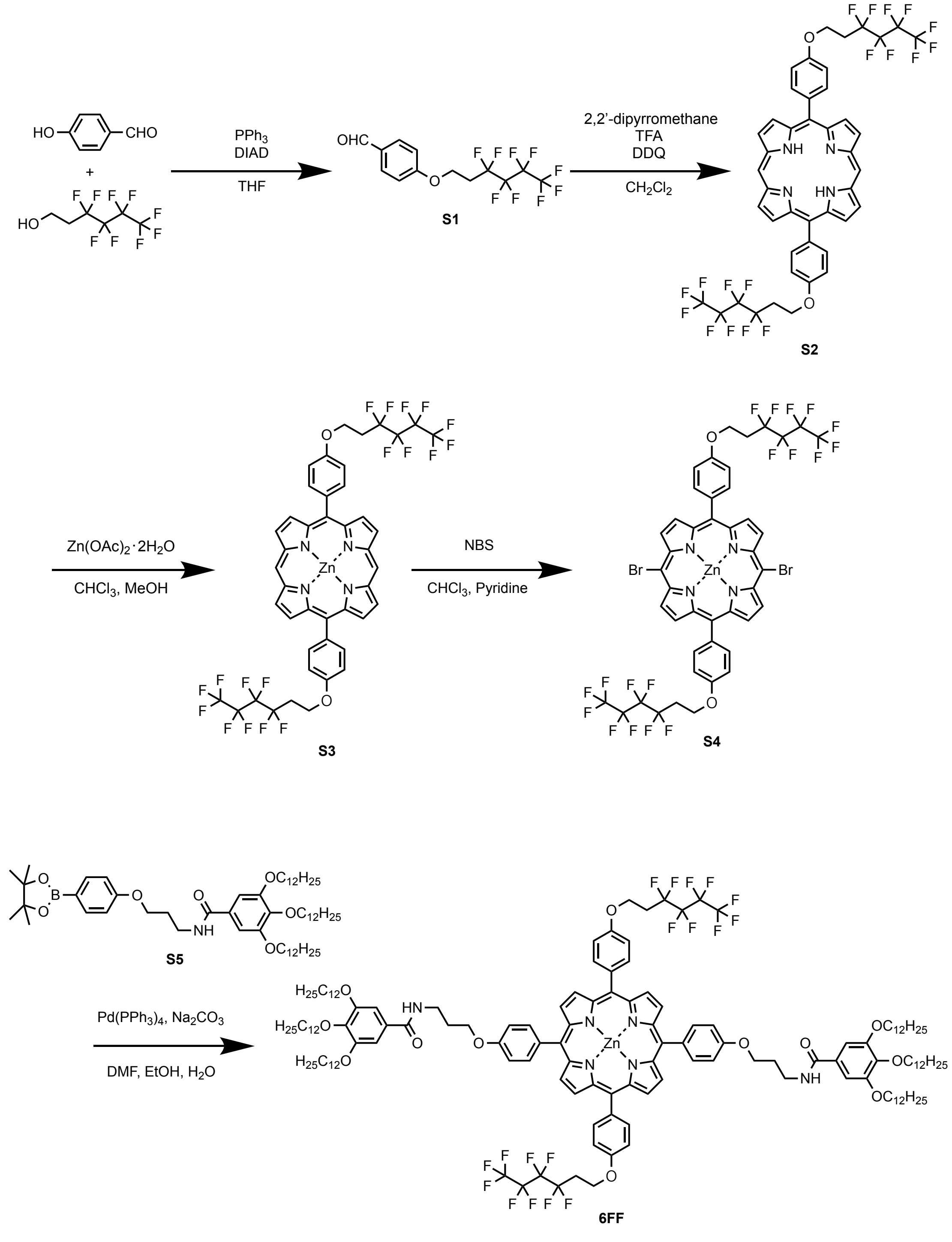

Synthesis of S1.

To a solution of 4-hydroxybenzaldehyde (2.79 g, 22.9 mmol), 1*H*,1*H*,2*H*,2*H*-nonafluoro-1-hexanol (1.9 mL, 11.4 mmol), triphenylphosphine (PPh_3) (5.95 g, 22.7 mmol) in tetrahydrofuran (THF) (25 mL), diisopropyl azodicarboxylate (DIAD) (4.8 mL, 24.4 mmol) was added at 0 °C under argon atmosphere. The solution was stirred at 40 °C for 23 h, cooled to room temperature, and diluted with ethyl acetate. The mixture was washed with water and dried over sodium sulfate anhydrous. The solvent was evaporated, and the solid residue was purified through column chromatography (silica gel, CH_2Cl_2 : hexane = 1:1) to provide **S1** as a white solid (260 mg, yield: 6 %).

^{1}H NMR ($CDCl_3$, 298 K) δ 2.62-2.74 (2H, m, CF_3-$(CF_2)_2$-CF_2-CH_2-CH_2-O-), 4.36 (2H, t, J = 7.2 Hz, CF_3-$(CF_2)_2$-CF_2-CH_2-CH_2-O-), 7.02 (2H, d, J = 8.8 Hz, Ar-*H*), 7.86 (2H, d, J = 8.8 Hz, Ar-*H*), 9.91 (1H, s, -C*H*O).; ^{19}F NMR ($CDCl_3$, 298 K) δ −81.3 (3F, br. m, CF_3-$(CF_2)_2$-CF_2-CH_2-CH_2-O-), −113.8 (2F, br. m, CF_3-$(CF_2)_2$-CF_2-CH_2-CH_2-O-), −124.8 (2F, br. m, CF_3-$(CF_2)_2$-CF_2-CH_2-CH_2-O-), −126.3 (2F, br. m, CF_3-$(CF_2)_2$-CF_2-CH_2-CH_2-O-).; MALDI-TOF mass (α-cyano-4-hydroxycinnamic acid): calcd. for $C_{13}H_9F_9O_2$: 368.05; found: 368.89.

Synthesis of S2

To a solution of 2,2'-dipyrromethane (149 mg, 1.02 mmol) and **S1** (357 mg, 0.969 mmol) in CH_2Cl_2 (248 mL), trifluoroacetic acid (TFA) (8.0 μL, 0.104 mmol) was added under argon atmosphere. The solution was stirred at room temperature for 18 h in the dark. To this mixture, 2,3-dichloro-5,6-dicyano-1,4-benzoquinone (DDQ) (308 mg, 1.36 mmol) was added, and then, the mixture was stirred at room temperature for 1 h in the dark. After adding triethylamine (0.78 mL) to this solution, the resulting mixture was subjected to flash column chromatography (silica gel, CH_2Cl_2) and reprecipitation (methanol) to yield compound **S2** as a purple solid (156 mg, yield: 32%).

^{1}H NMR ($CDCl_3$, 298 K) δ −3.11 (2H, br, inner-H), 2.82-2.95 (4H, m, CF_3-$(CF_2)_2$-CF_2-CH_2-CH_2-O-), 4.62 (4H, t, J = 6.4 Hz, CF_3-$(CF_2)_2$-CF_2-CH_2-CH_2-O-), 7.36 (4H, d, J = 8.8 Hz, Ar-*H*), 8.21 (4H, d, J = 8.8 Hz, Ar-*H*), 9.10 (4H, d, J = 5.0 Hz, β-pyrrole), 9.41 (4H, d, J = 5.0 Hz, β-pyrrole), 10.33 (2H, s, *meso*-H).; ^{19}F NMR ($CDCl_3$, 298 K) δ −81.2 (3F, br. m, CF_3-$(CF_2)_2$-CF_2-CH_2-CH_2-O-), −113.6 (2F, br. m, CF_3-$(CF_2)_2$-CF_2-CH_2-CH_2-O-), −124.6 (2F, br. m, CF_3-$(CF_2)_2$-CF_2-CH_2-CH_2-O-), −126.1 (2F, br. m, CF_3-$(CF_2)_2$-CF_2-CH_2-CH_2-O-).; MALDI-TOF mass (dithranol): calcd. for $C_{44}H_{28}F_{18}N_4O_2Zn$: 986.19; found: 986.88.

Synthesis of S3

A solution of compound **S2** (139 mg, 0.141 mmol) in $CHCl_3$ (90 mL) was refluxed with stirring, and then, zinc acetate dihydrate (362 mg, 1.65 mmol) in methanol (14 mL) was added to the solution. After stirring for 5 h, the solvent was evaporated in vacuo to dryness, and the residue was purified through column chromatography (silica gel, $CHCl_3$) and reprecipitation (methanol) to yield compound **S3** as a pink solid (131 mg, 84%).

^{1}H NMR ($CDCl_3$, 298 K) δ 2.85-2.94 (4H, m, CF_3-$(CF_2)_2$-CF_2-CH_2-CH_2-O-), 4.63 (4H, t, J = 6.4 Hz, CF_3-$(CF_2)_2$-CF_2-CH_2-CH_2-O-), 7.35 (4H, d, J = 8.6 Hz, Ar-H), 8.21 (4H, d, J = 8.6 Hz, Ar-H), 9.17 (4H, d, J = 4.4 Hz, β-pyrrole), 9.47 (4H, d, J = 4.4 Hz, β-pyrrole), 10.35 (2H, s, *meso*-H).; ^{19}F NMR ($CDCl_3$, 298 K) δ −81.2 (3F, br. m, CF_3-$(CF_2)_2$-CF_2-CH_2-CH_2-O-), −113.6 (2F, br. m, CF_3-$(CF_2)_2$-CF_2-CH_2-CH_2-O-), −124.6 (2F, br. m, CF_3-(CF_2)$_2$-CF_2-CH_2-CH_2-O-), −126.1 (2F, br. m, CF_3-(CF_2)$_2$-CF_2-CH_2-CH_2-O-).; MALDI-TOF mass (dithranol): calcd. for $C_{44}H_{26}F_{18}N_4O_2Zn$: 1048.11; found: 1047.80.

Synthesis of S4

A solution of compound **S3** (115 mg, 110 μmol) in $CHCl_3$ (24 mL) and pyridine (0.1 mL) was cooled to 0 °C in an ice bath, and *N*-bromosuccinimide (NBS) (40.2 mg, 226 μmol) was added with stirring in the dark. After 2 h, the cooling bath was removed and the mixture was stirred at room temperature for another 1 h in the dark. The reaction was quenched with acetone (10 mL), and the solvent was evaporated in vacuo. The residue was purified through column chromatography (silica gel, $CHCl_3$: pyridine = 400 : 1) and reprecipitation (methanol) to provide **S4** as a purple solid (129 mg, 98%).

^{1}H NMR ($CDCl_3$/pyridine-d_5, 95:5, 298 K) δ 2.79-2.92 (4H, m, CF_3-$(CF_2)_2$-CF_2-CH_2-CH_2-O-), 4.57 (4H, t, J = 6.8 Hz, CF_3-$(CF_2)_2$-CF_2-CH_2-CH_2-O-), 7.27 (4H, d, J = 8.6 Hz, Ar-H), 8.06 (4H, d, J = 8.6 Hz, Ar-H), 8.88 (4H, d, J = 5.0 Hz, β-pyrrole), 9.66 (4H, d, J = 5.0 Hz, β-pyrrole).; ^{19}F NMR ($CDCl_3$/pyridine-d_5, 95:5, 298 K) δ −81.2 (3F, br. m, CF_3-$(CF_2)_2$-CF_2-CH_2-CH_2-O-), −113.6 (2F, br. m, CF_3-$(CF_2)_2$-CF_2-CH_2-CH_2-O-), −124.6 (2F, br. m, CF_3-(CF_2)$_2$-CF_2-CH_2-CH_2-O-), −126.1 (2F, br. m, CF_3-(CF_2)$_2$-CF_2-CH_2-CH_2-O-).; MALDI-TOF mass (dithranol): calcd. for $C_{44}H_{24}Br_2F_{18}N_4O_2Zn$: 1205.93; found: 1205.60.

Synthesis of 6FF

To a solution of compound **S4** (117 mg, 97.1 μmol), **S5** (202 mg, 216 μmol), sodium carbonate (56.5 mg, 533 μmol) in *N*,*N*-dimethylformamide (DMF) (15 mL), ethanol (2 mL) and water (2 mL) mixed solvent, tetrakis(triphenylphosphine)palladium(0) (4.22 mg, 3.65 μmol) was added under argon atmosphere. The solution was stirred at 90 °C for 3 h, cooled to room temperature, and diluted with $CHCl_3$. The mixture was washed with ammonium chloride aqueous solution, water, and brine, and dried over magnesium sulfate anhydrous. The solvent was evaporated, and the solid residue was purified through column chromatography (silica gel, $CHCl_3$), size exclusion chromatography (Bio beads SX-1, CH_2Cl_2) and reprecipitation (methanol) to provide **6FF** as a purple solid (66 mg, yield: 59 %).

m.p. = 183-185 °C; ^{1}H NMR ($CDCl_3$, 298 K) δ 0.81-0.87 (18H, m, CH_3-), 1.07-1.51 (108H, m, CH_3-(CH_2)$_9$-CH_2-CH_2-O-), 1.69-1.83 (12H, m, CH_3-$(CH_2)_9$-CH_2-CH_2-O-), 2.27-2.33 (4H, m, -NH-CH_2-CH_2-CH_2-O-), 2.79-2.92 (4H, m, CF_3-$(CF_2)_2$-CF_2-CH_2-CH_2-O-), 3.76-3.80 (4H, m, -NH-CH_2-CH_2-CH_2-O-), 3.97-4.06 (12H, m, CH_3-$(CH_2)_9$-CH_2-CH_2-O-), 4.42 (4H, t, J = 5.2 Hz, -NH-CH_2-CH_2-CH_2-O-), 4.59 (4H, t, J = 6.4 Hz, CF_3-$(CF_2)_2$-CF_2-CH_2-CH_2-O-), 6.66 (2H, t, J = 5.2 Hz, -NH-CH_2-CH_2-CH_2-O-), 7.02 (4H, s, Ar-H), 7.29 (8H, m, Ar-H), 8.12 (4H, d, J = 8.4 Hz, Ar-H), 8.14 (4H,

d, J = 8.4 Hz, Ar-H), 8.95 (8H, s, β-pyrrole).; ^{13}C NMR ($CDCl_3$, 298 K) δ 14.0, 22.62, 22.65, 26.08, 26.09, 29.18, 29.26, 29.36, 29.40, 29.56, 29.65, 29.71, 30.32, 31.84, 31.90, 38.46, 60.29, 67.24, 69.44, 73.50, 105.68, 112.55, 112.60, 120.45, 120.53, 129.45, 131.83, 135.46, 135.49, 135.76, 136.12, 141.24, 150.42, 150.45, 153.11, 157.76, 158.25, 167.41; ^{19}F NMR ($CDCl_3$, 298 K) δ −81.2 (3F, br. m, CF_3-$(CF_2)_2$-CF_2-CH_2-CH_2-O-), −113.7 (2F, br. m, CF_3-$(CF_2)_2$-CF_2-CH_2-CH_2-O-), −124.6 (2F, br. m, CF_3-(CF_2)$_2$-CF_2-CH_2-CH_2-O-), −126.1 (2F, br. m, CF_3-(CF_2)$_2$-CF_2-CH_2-CH_2-O-).; MALDI-TOF mass (dithranol): calcd. for $C_{148}H_{200}F_{18}N_6O_{12}Zn$: 2660.43; found: 2659.73; Elemental analysis: calcd. for $C_{148}H_{200}F_{18}N_6O_{12}Zn$ (%): C, 66.76; H, 7.57; F, 12.84; N, 3.16; O, 7.21; Zn, 2.46: found: C, 66.69; H, 7.59; N, 3.23.

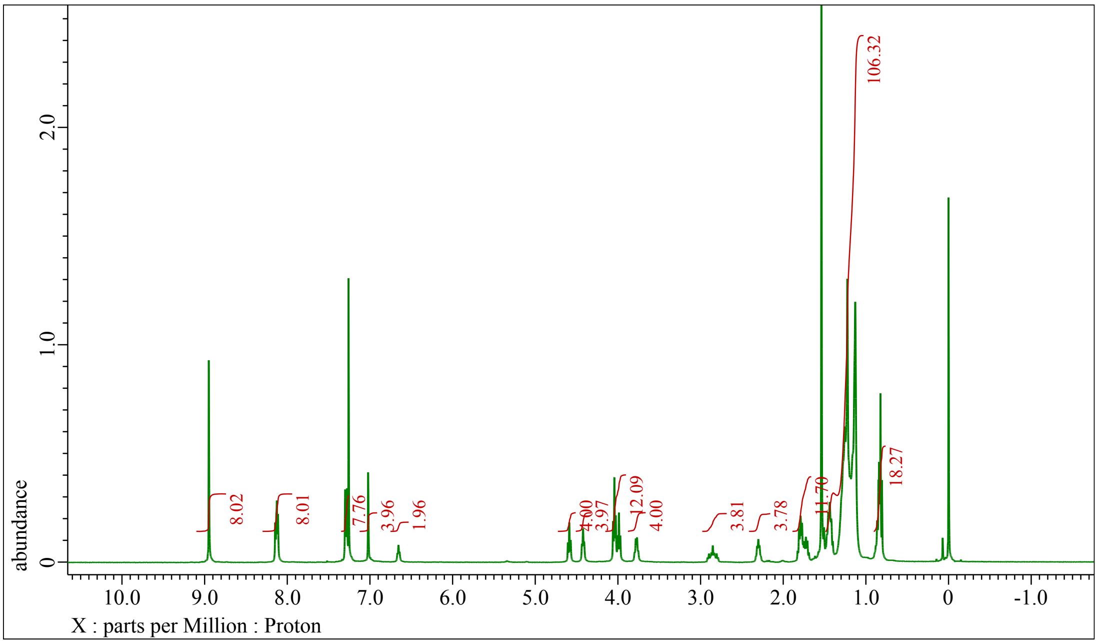

Supplementary Figure 1
^{1}H NMR (400 MHz) spectrum of **6FF** in $CDCl_3$ at 298 K

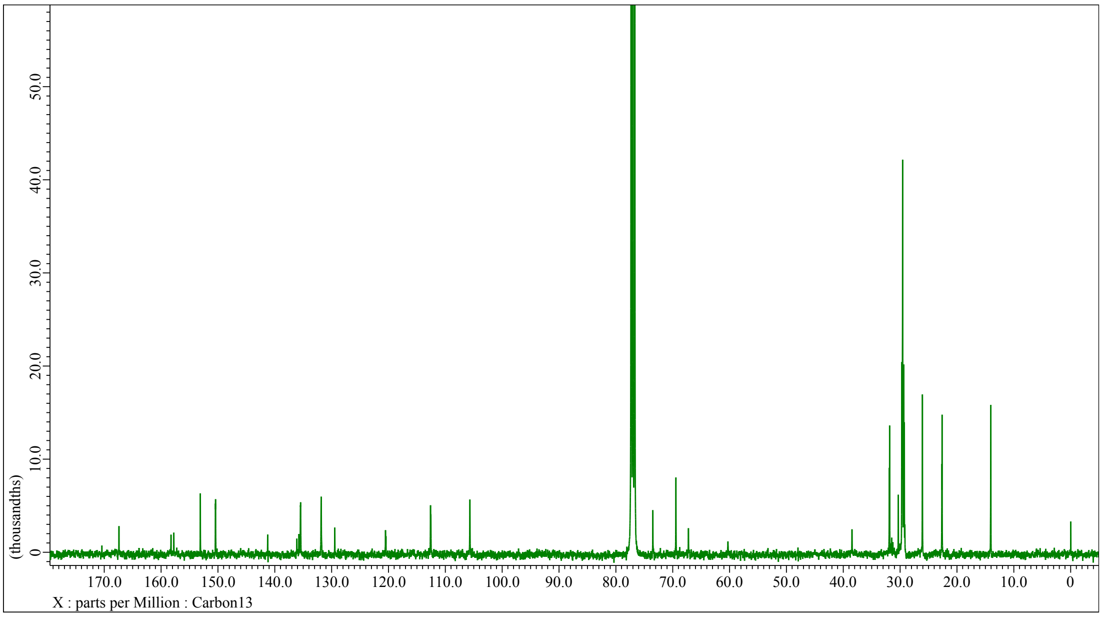

Supplementary Figure 2
^{13}C NMR (100 MHz) spectrum of **6FF** in $CDCl_3$ at 298 K

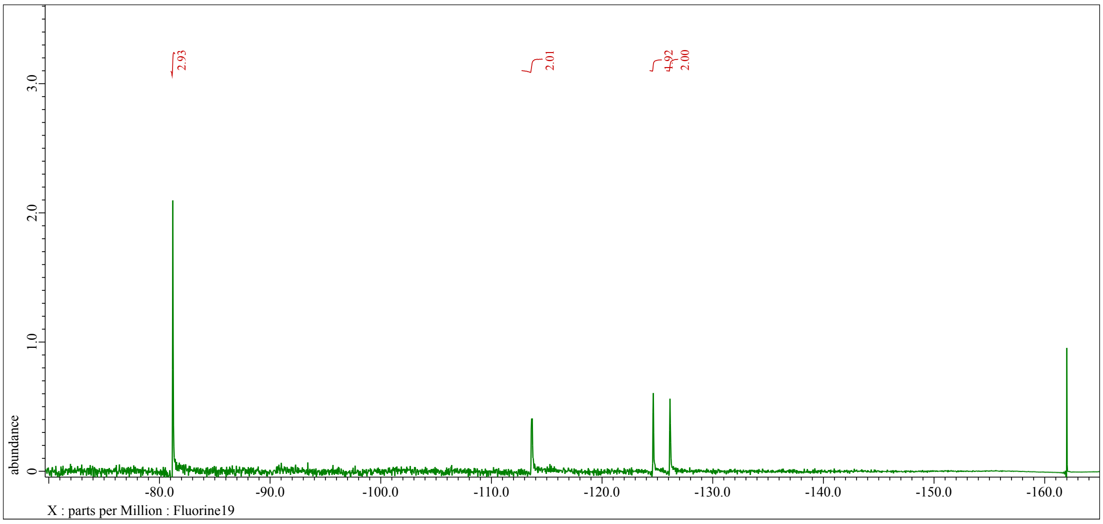

Supplementary Figure 3
^{19}F NMR (376 MHz) spectrum of **6FF** in $CDCl_3$ at 298 K

Synthesis of 6FH

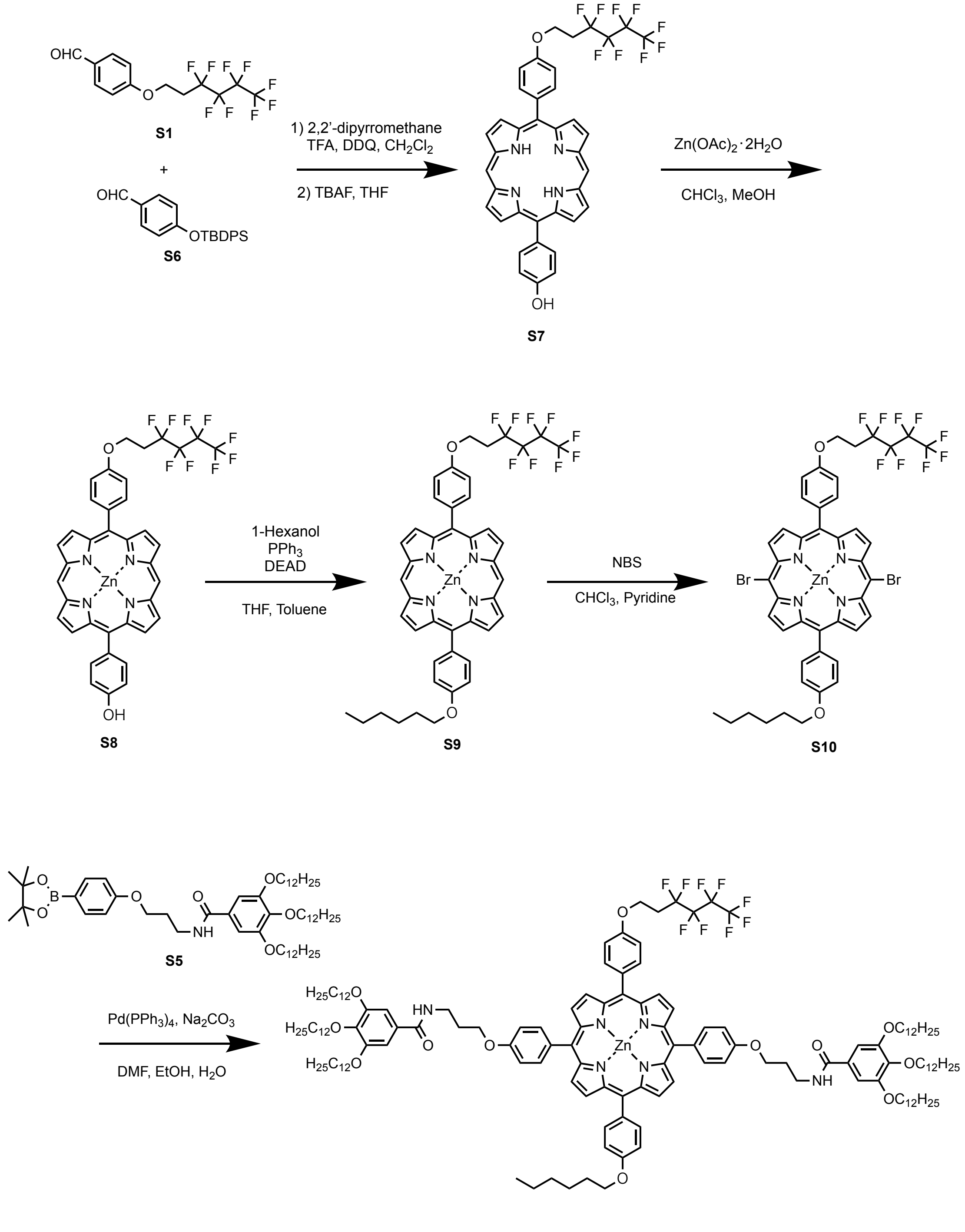

Synthesis of S7

To a solution of 2,2'-dipyrromethane (198 mg, 1.35 mmol), **S1** (234 mg, 636 μmol) and **S6** (230 mg, 638 μmol) in CH_2Cl_2 (324 mL), TFA (10 μL, 0.131 mmol) was added under argon atmosphere. The solution was stirred at room temperature for 16 h in the dark. To this mixture, DDQ (409 mg, 1.80 mmol) was added, and then, the mixture was stirred at room temperature for 2 h in the dark. After adding triethylamine (1.0 mL) to this solution, the resulting mixture was subjected to flash column chromatography (silica gel, CH_2Cl_2) and reprecipitation (methanol) to provide porphyrin derivatives as a purple solid (316 mg). To a solution of the porphyrin derivatives (316 mg) in THF (163 mL), a THF solution of tetrabutylammonium fluoride (TBAF) (1 M, 0.5 mL) was added, and the resultant solution was stirred at room temperature for 1 h and diluted with ethyl acetate. The mixture was washed with water and brine, and dried over magnesium sulfate anhydrous. The solvent was evaporated, and the solid residue was purified through column chromatography (silica gel, $CHCl_3$: acetone = 20 : 1) to provide **S7** as a purple solid (76 mg, yield: 8.1 %).

^{1}H NMR ($CDCl_3$, 298 K) δ −3.09 (2H, br, inner-H), 2.84-2.95 (2H, m, CF_3-$(CF_2)_2$-CF_2-CH_2-CH_2-O-), 4.62 (2H, t, J = 6.4 Hz, CF_3-$(CF_2)_2$-CF_2-CH_2-CH_2-O-), 7.34-7.39 (4H, m, Ar-*H*), 8.09 (2H, d, J = 8.8 Hz, Ar-*H*), 8.21 (2H, d, J = 8.8 Hz, Ar-*H*), 8.94 (1H, s, *H*O-), 9.09 (2H, d, J = 4.8 Hz, β-pyrrole), 9.16 (2H, d, J = 4.4 Hz, β-pyrrole), 9.39-9.41 (4H, m, β-pyrrole), 10.31 (2H, s, *meso*-H).; ^{19}F NMR ($CDCl_3$, 298 K) δ −81.2 (3F, br. m, CF_3-$(CF_2)_2$-CF_2-CH_2-CH_2-O-), −113.7 (2F, br. m, CF_3-$(CF_2)_2$-CF_2-CH_2-CH_2-O-), −124.6 (2F, br. m, CF_3-(CF_2)$_2$-CF_2-CH_2-CH_2-O-), −126.1 (2F, br. m, CF_3-(CF_2)$_2$-CF_2-CH_2-CH_2-O-).; MALDI-TOF mass (dithranol): calcd. for $C_{38}H_{25}F_9N_4O_2Zn$: 740.18; found: 740.89.

Synthesis of S8

A solution of compound **S7** (71 mg, 95.4 μmol) in $CHCl_3$ (57 mL) was refluxed with stirring, and then zinc acetate dihydrate (209 mg, 0.95 mmol) in methanol (9 mL) was added to the solution. After stirring for 3 h, the solvent was evaporated in vacuo to dryness, and the residue was purified through column chromatography (silica gel, $CHCl_3$: acetone = 20 : 1) and reprecipitation (methanol) to yield compound **S8** as a pink solid (94 mg, quant).

^{1}H NMR ($CDCl_3$/pyridine-d_5, 95:5, 298 K) δ 2.80-2.93 (2H, m, CF_3-$(CF_2)_2$-CF_2-CH_2-CH_2-O-), 4.60 (2H, t, J = 7.2 Hz, CF_3-$(CF_2)_2$-CF_2-CH_2-CH_2-O-), 7.30-7.33 (4H, m, Ar-*H*), 8.09 (2H, d, J = 8.6 Hz, Ar-*H*), 8.18 (2H, d, J = 8.6 Hz, Ar-*H*), 9.08 (2H, d, J = 4.8 Hz, β-pyrrole), 9.17 (2H, d, J = 4.6 Hz, β-pyrrole), 9.36 (2H, d, J = 4.8 Hz, β-pyrrole), 9.54 (2H, d, J = 4.6 Hz, β-pyrrole), 10.19 (2H, s, *meso*-H).; ^{19}F NMR ($CDCl_3$/pyridine-d_5, 95:5, 298 K) δ −81.2 (3F, br. m, CF_3-$(CF_2)_2$-CF_2-CH_2-CH_2-O-), −113.6 (2F, br. m, CF_3-$(CF_2)_2$-CF_2-CH_2-CH_2-O-), −124.6 (2F, br. m, CF_3-(CF_2)$_2$-CF_2-CH_2-CH_2-O-), −126.1 (2F, br. m, CF_3-(CF_2)$_2$-CF_2-CH_2-CH_2-O-).; MALDI-TOF mass (dithranol): calcd. for $C_{38}H_{23}F_9N_4O_2Zn$: 802.10; found: 801.79.

Synthesis of S9.

To a solution of compound **S8** (85 mg, 106 μmol), *n*-hexanol (26 μL, 212 μmol), and PPh_3 (53 mg, 200 μmol) in THF (0.9 mL), 2.2 M toluene solution of diethyl

azodicarboxylate (DEAD) (100 μL, 220 μmol) was added at 0 °C under argon atmosphere. The solution was stirred at 65 °C overnight in the dark, cooled to room temperature, and diluted with ethyl acetate. The mixture was washed with water, brine, and dried over sodium sulfate anhydrous. The solvent was evaporated, and the solid residue was purified through column chromatography (silica gel, $CHCl_3$) to provide **S9** as a purple solid (42 mg, yield: 45 %).

^1H NMR ($CDCl_3$, 298 K) δ 1.01 (3H, t, J = 7.6 Hz, CH_3-CH_2-CH_2-CH_2-CH_2-CH_2-O-), 1.45-1.54 (4H, m, CH_3-CH_2-CH_2-CH_2-CH_2-CH_2-O-), 1.63-1.71 (2H, m, CH_3-CH_2-CH_2-CH_2-CH_2-CH_2-O-), 1.99-2.06 (2H, m, CH_3-CH_2-CH_2-CH_2-CH_2-CH_2-O-), 2.82-2.95 (2H, m, CF_3-$(CF_2)_2$-CF_2-CH_2-CH_2-O-), 4.30 (2H, t, J = 7.2 Hz, CH_3-CH_2-CH_2-CH_2-CH_2-CH_2-O-), 4.62 (2H, t, J = 7.2 Hz, CF_3-$(CF_2)_2$-CF_2-CH_2-CH_2-O-), 7.33 (2H, d, J = 8.4 Hz, Ar-H), 7.34 (2H, d, J = 8.8 Hz, Ar-H), 8.16 (2H, d, J = 8.8 Hz, Ar-H), 8.19 (2H, d, J = 8.4 Hz, Ar-H), 9.15 (2H, d, J = 4.4 Hz, β-pyrrole), 9.19 (2H, d, J = 4.8 Hz, β-pyrrole), 9.437 (2H, d, J = 4.4 Hz, β-pyrrole), 9.439 (2H, d, J = 4.8 Hz, β-pyrrole), 10.31 (2H, s, *meso*-H).; ^{19}F NMR ($CDCl_3$, 298 K) δ −81.2 (3F, br. m, CF_3-$(CF_2)_2$-CF_2-CH_2-CH_2-O-), −113.6 (2F, br. m, CF_3-$(CF_2)_2$-CF_2-CH_2-CH_2-O-), −124.6 (2F, br. m, CF_3-$(CF_2)_2$-CF_2-CH_2-CH_2-O-), −126.2 (2F, br. m, CF_3-$(CF_2)_2$-CF_2-CH_2-CH_2-O-).; MALDI-TOF mass (dithranol): calcd. for $C_{44}H_{35}F_9N_4O_2Zn$: 886.19; found: 885.79.

Synthesis of S10

A solution of compound **S9** (41.2 mg, 46.3 μmol) in $CHCl_3$ (12 mL) and pyridine (45 μL) was cooled to 0 °C in an ice bath, and NBS (17.4 mg, 97.5 μmol) was added with stirring. The cooling bath was removed, and the mixture was stirred at room temperature for 2 h. The reaction was quenched with acetone (5 mL), and the solvent was evaporated in vacuo. The residue was purified through column chromatography (silica gel, $CHCl_3$: pyridine = 400 : 1) and reprecipitation (methanol) to provide **S10** as a purple solid (32 mg, 66%).

^1H NMR ($CDCl_3$/pyridine-d_5, 95:5, 298 K) δ 1.01 (3H, t, J = 6.8 Hz, CH_3-CH_2-CH_2-CH_2-CH_2-CH_2-O-), 1.43-1.52 (4H, m, CH_3-CH_2-CH_2-CH_2-CH_2-CH_2-O-), 1.62-1.69 (2H, m, CH_3-CH_2-CH_2-CH_2-CH_2-CH_2-O-), 1.97-2.04 (2H, m, CH_3-CH_2-CH_2-CH_2-CH_2-CH_2-O-), 2.80-2.93 (2H, m, CF_3-$(CF_2)_2$-CF_2-CH_2-CH_2-O-), 4.27 (2H, t, J = 6.4 Hz, CH_3-CH_2-CH_2-CH_2-CH_2-CH_2-O-), 4.59 (2H, t, J = 7.2 Hz, CF_3-$(CF_2)_2$-CF_2-CH_2-CH_2-O-), 7.26-7.29 (4H, m, Ar-H), 8.04 (2H, d, J = 8.6 Hz, Ar-H), 8.07 (2H, d, J = 8.6 Hz, Ar-H), 8.87 (2H, d, J = 4.8 Hz, β-pyrrole), 8.91 (2H, d, J = 4.6 Hz, β-pyrrole) 9.64 (2H, d, J = 4.8 Hz, β-pyrrole), 9.65 (2H, d, J = 4.6 Hz, β-pyrrole).; ^{19}F NMR ($CDCl_3$/pyridine-d_5, 95:5, 298 K) δ −81.2 (3F, br. m, CF_3-$(CF_2)_2$-CF_2-CH_2-CH_2-O-), −113.7 (2F, br. m, CF_3-$(CF_2)_2$-CF_2-CH_2-CH_2-O-), −124.6 (2F, br. m, CF_3-$(CF_2)_2$-CF_2-CH_2-CH_2-O-), −126.1 (2F, br. m, CF_3-$(CF_2)_2$-CF_2-CH_2-CH_2-O-).; MALDI-TOF mass (dithranol): calcd. for $C_{44}H_{33}Br_2F_9N_4O_2Zn$: 1044.01; found: 1043.44.

Synthesis of 6FH

To a solution of compound **S10** (30.3 mg, 29.0 μmol), **S5** (108 mg, 116 μmol), sodium carbonate (19.1 mg, 108 μmol) in DMF (4 mL), ethanol (0.65 mL) and water (0.65 mL) mixed solvent, tetrakis(triphenylphosphine)palladium(0) (1.14 mg, 0.99

μmol) was added under argon atmosphere. The solution was stirred at 90 °C for 6 h, cooled to room temperature, and diluted with ethyl acetate. The mixture was washed with ammonium chloride aqueous solution and water, and dried over sodium sulfate anhydrous. The solvent was evaporated, and the solid residue was purified through column chromatography (silica gel, $CHCl_3$: acetone = 20 : 1) and recycling gel permeation chromatography ($CHCl_3$) to provide **6FH** as a purple solid (38 mg, yield: 53 %).

m.p. = 182-185 °C; ^1H NMR ($CDCl_3$, 298 K) δ 0.81-0.88 (18H, m, CH_3-), 0.99 (3H, t, J = 7.2 Hz, CH_3-CH_2-CH_2-CH_2-CH_2-CH_2-O-), 1.09-1.33 (96H, m, CH_3-$(CH_2)_8$-CH_2-CH_2-CH_2-O-), 1.40-1.49 (16H, m, CH_3-$(CH_2)_8$-CH_2-CH_2-CH_2-O-, CH_3-CH_2-CH_2-CH_2-CH_2-CH_2-O-), 1.63-1.83 (14H, m, CH_3-$(CH_2)_8$-CH_2-CH_2-CH_2-O-, CH_3-CH_2-CH_2-CH_2-CH_2-CH_2-O-), 1.96-2.03 (2H, m, CH_3-CH_2-CH_2-CH_2-CH_2-CH_2-O-), 2.27-2.33 (4H, m, -NH-CH_2-CH_2-CH_2-O-), 2.79-2.92 (2H, m, CF_3-$(CF_2)_2$-CF_2-CH_2-CH_2-O-), 3.75-3.79 (4H, m, -NH-CH_2-CH_2-CH_2-O-), 3.99 (4H, t, J = 6.4 Hz, CH_3-$(CH_2)_8$-CH_2-CH_2-CH_2-O-), 4.04 (8H, t, J = 7.2 Hz, CH_3-$(CH_2)_8$-CH_2-CH_2-CH_2-O-), 4.26 (2H, t, J = 6.4 Hz, CH_3-CH_2-CH_2-CH_2-CH_2-CH_2-O-), 4.42 (4H, t, J = 5.6 Hz, -NH-CH_2-CH_2-CH_2-O-), 4.59 (2H, t, J = 6.8 Hz, CF_3-$(CF_2)_2$-CF_2-CH_2-CH_2-O-), 6.67 (2H, t, J = 5.6 Hz, -N*H*-CH_2-CH_2-CH_2-O-), 7.01 (4H, s, Ar-*H*), 7.27-7.30 (8H, m, Ar-*H*), 8.04-8.15 (8H, m, Ar-*H*), 8.94 (6H, d, J = 4.8 Hz, β-pyrrole), 8.98 (2H, d, J = 4.8 Hz, β-pyrrole).; ^{13}C NMR ($CDCl_3$, 298 K) δ 14.07, 22.64, 22.67, 25.93, 26.11, 29.19, 29.29, 29.38, 29.58, 29.67, 29.73, 30.34, 31.76, 31.87, 31.92, 38.52, 60.31, 67.28, 68.35, 69.47, 73.52, 105.72, 112.56, 112.60, 120.33, 120.47, 121.02, 129.51, 131.78, 132.03, 135.04, 135.41, 135.51, 135.84, 136.18, 141.26, 150.40, 150.46, 150.59, 153.14, 157.76, 158.25, 158.86, 167.46.; ^{19}F NMR ($CDCl_3$, 298 K) δ −81.2 (3F, br. m, CF_3-$(CF_2)_2$-CF_2-CH_2-CH_2-O-), −113.7 (2F, br. m, CF_3-$(CF_2)_2$-CF_2-CH_2-CH_2-O-), −124.6 (2F, br. m, CF_3-$(CF_2)_2$-CF_2-CH_2-CH_2-O-), −126.2 (2F, br. m, CF_3-$(CF_2)_2$-CF_2-CH_2-CH_2-O-).; MALDI-TOF mass (dithranol): calcd. for $C_{148}H_{209}F_9N_6O_{12}Zn$: 2498.51; found: 2499.15.; Elemental analysis: calcd. for $C_{148}H_{209}F_9N_6O_{12}Zn$ (%): C, 71.09; H, 8.42; F, 6.84; N, 3.36; O, 7.68; Zn, 2.61: found: C, 71.28; H, 8.45; N, 3.50.

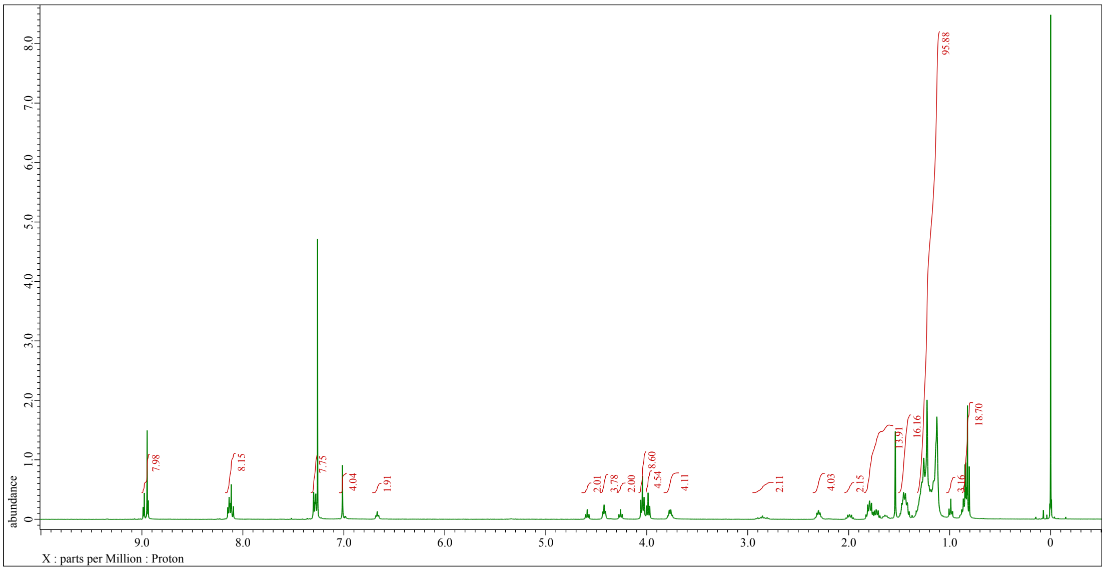

Supplementary Figure 4
^{1}H NMR (400 MHz) spectrum of **6FH** in $CDCl_3$ at 298 K

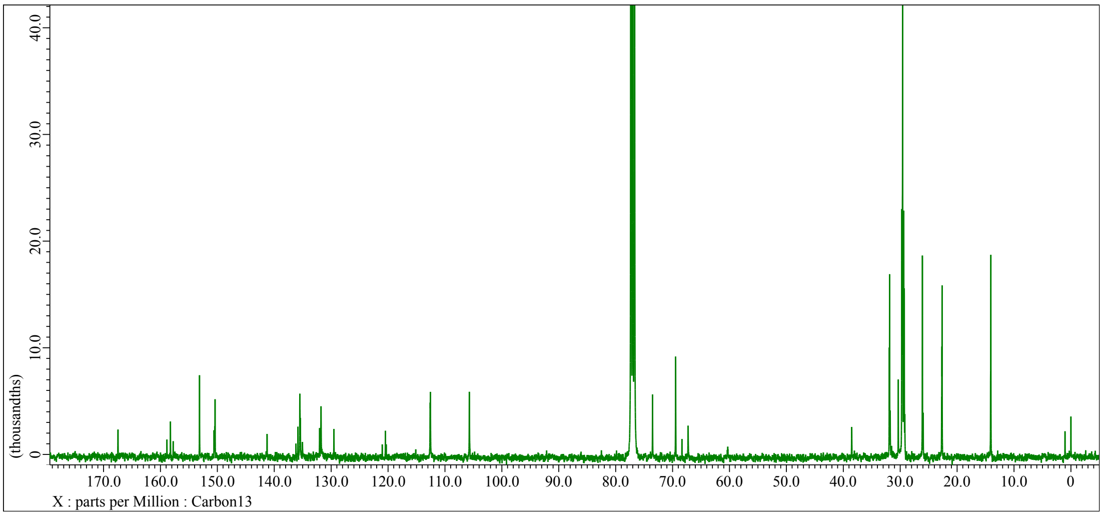

Supplementary Figure 5
^{13}C NMR (100 MHz)spectrum of **6FH** in $CDCl_3$ at 298 K

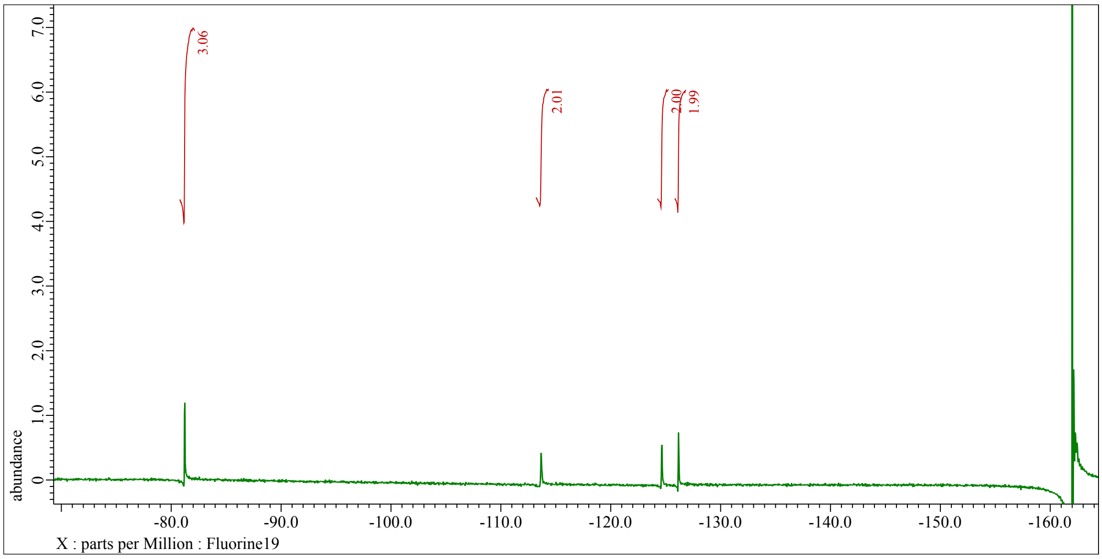

Supplementary Figure 6
^{19}F NMR (376 MHz) spectrum of **6FH** in $CDCl_3$ at 298 K

3. Supplementary Figures and Tables

AFM images of self-assembled structures of 6FF

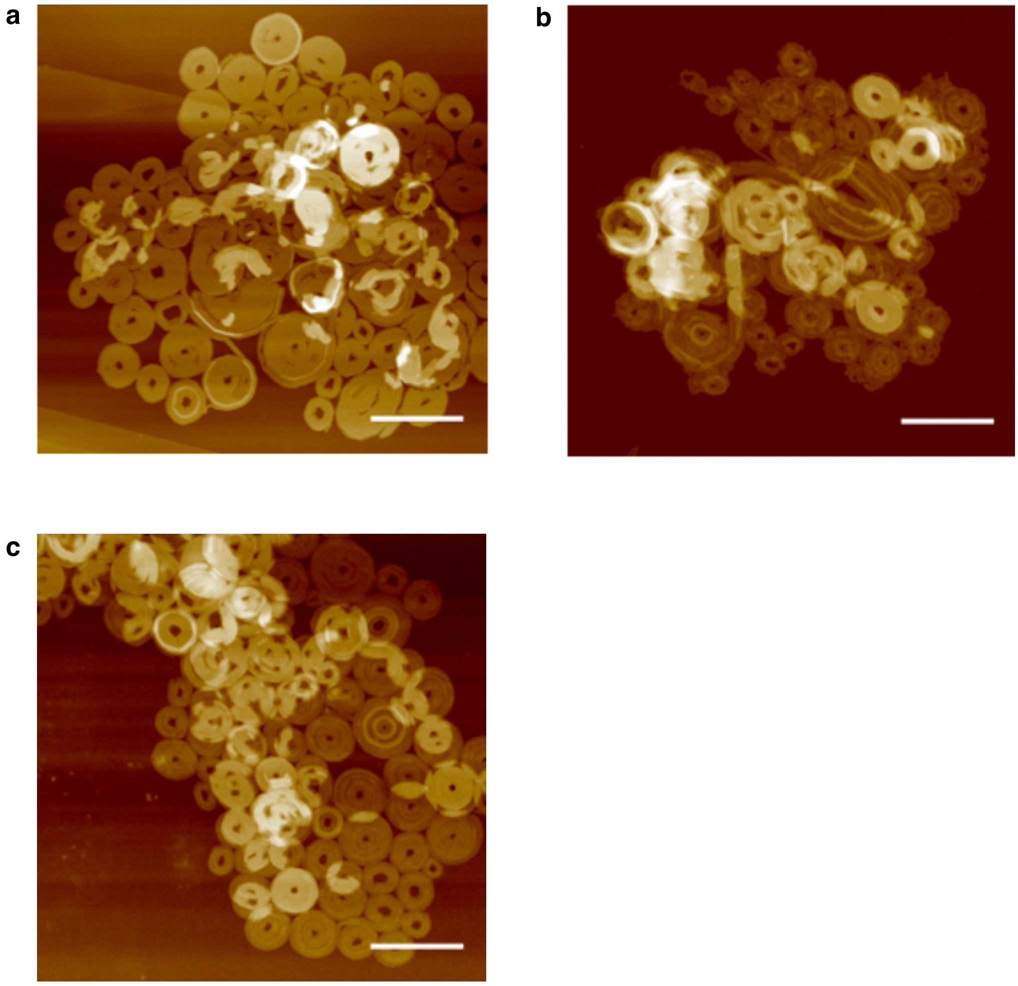

Supplementary Figure 7

AFM images of Archimedean spirals obtained by cooling a hot solution of **6FF** (12.5 μM) in dodecane and spin-coated on (a) HOPG, (b) mica, and (c) silicon wafer: scale bar = 400 nm.

AFM images of self-assembled structures of 6FF

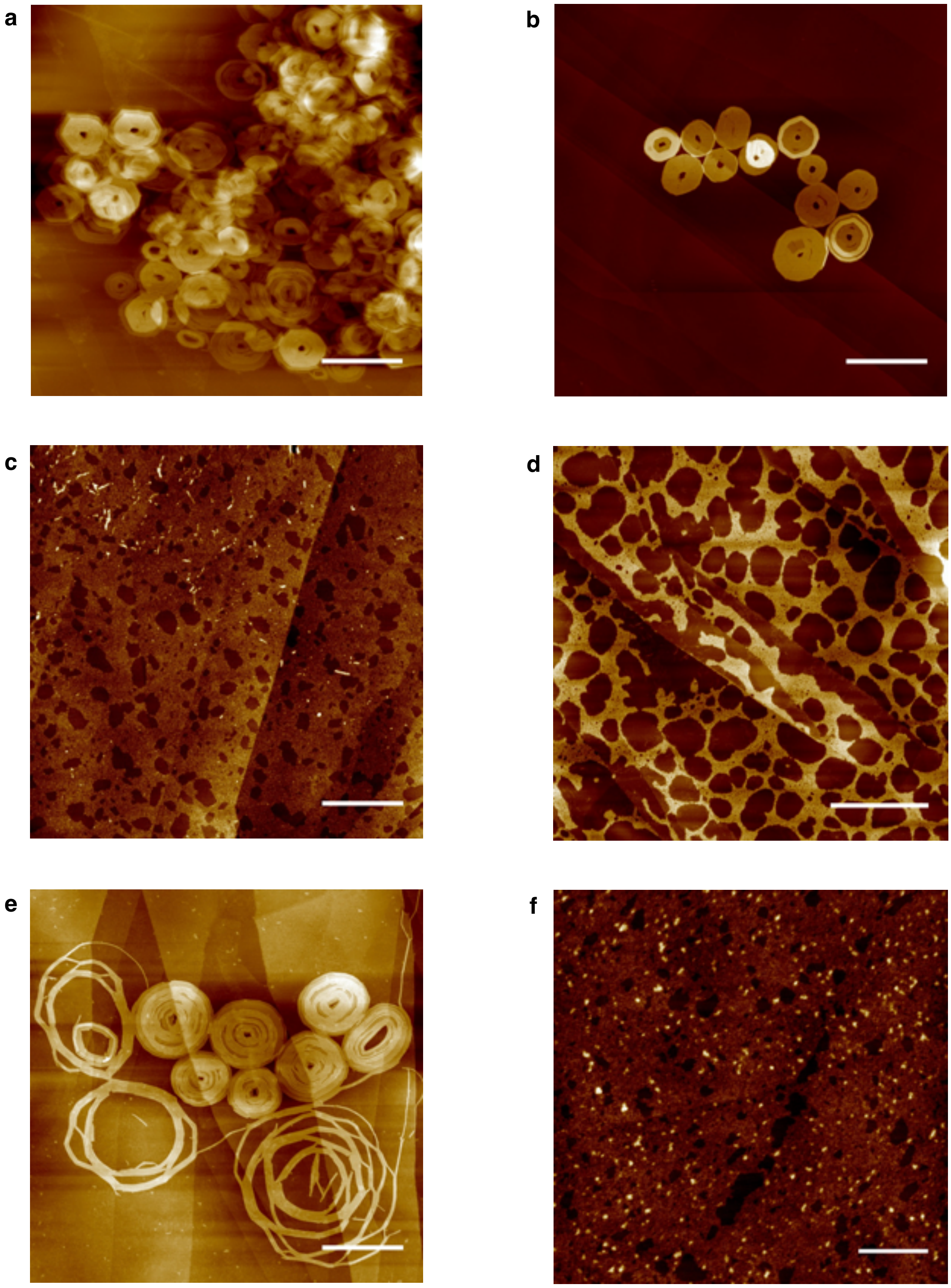

Supplementary Figure 8
AFM images of self-assembled structures obtained by cooling a hot solution of **6FF** (20 μM) in (a) hexane, (b) dodecane, (c) decalin, (d) toluene, (e) MCH, and (f) cyclohexane: scale bar = 500 nm (a-e) and 200 nm (f). The solutions were equilibrated for 5 hours at 298 K and spin-coated on HOPG substrates. Precipitates were observed in hexane, dodecane, and MCH.

Absorption spectra of 6FF in different solvents

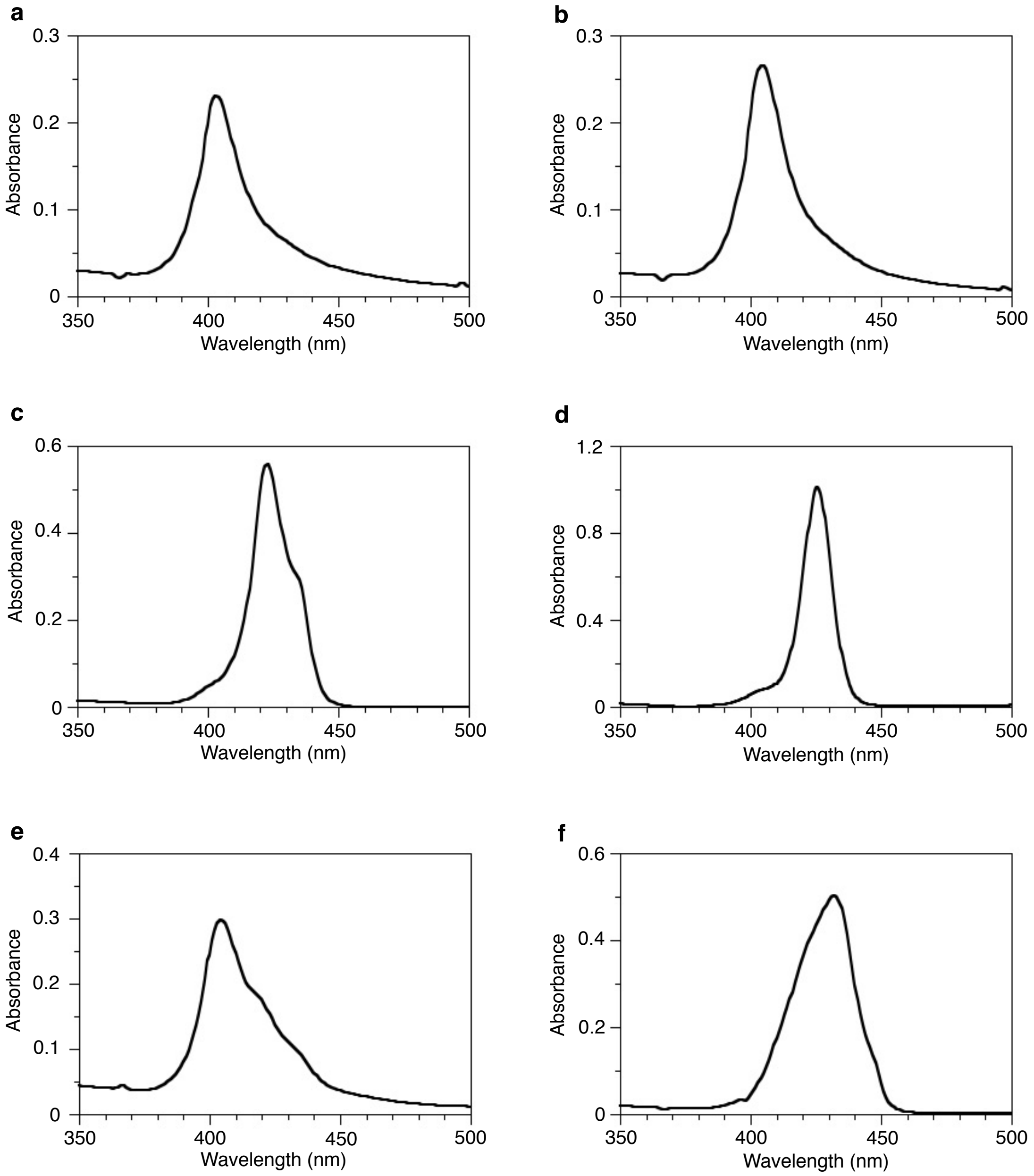

Supplementary Figure 9

Absorption spectra of **6FF** in (a) hexane, (b) dodecane, (c) decalin, (d) toluene, (e) MCH, and (f) cyclohexane: 20 μM at 298 K. The solutions were first heated to dissolve **6FF** and cooled down to 298 K, then equilibrated for 5 hours. Precipitates were observed in hexane, dodecane, and MCH; in such cases, precipitates were suspended by shaking the solution prior to the measurements.

Structural parameters of Archimedean spirals of 6FF

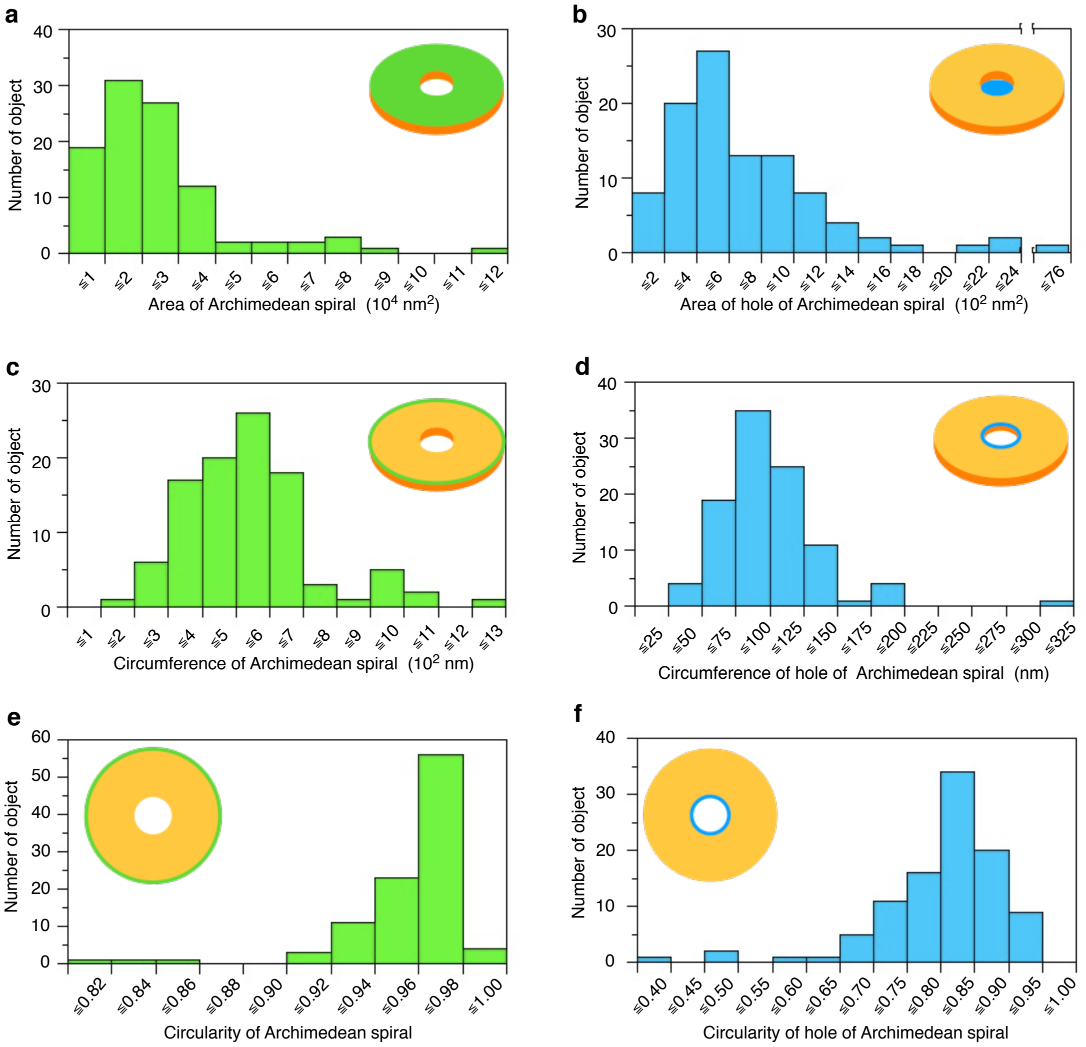

Supplementary Figure 10

Histograms of structural parameters of Archimedean spirals (green) and their holes (blue) formed from **6FF**: (a,b) area (A), (c,d) circumference (B), and (e,f) circularity (C); The circularity value is given by $C = 4\pi A/(B)^2$. The data were collected by tracing 100 objects in AFM images.

Supplementary Table 1

	Archimedean spirals	holes
Average area (nm^2)	24000	740
Circumference (nm)	540	100
Circularity	0.96	0.80

<u>Molecular model of 6FF</u>

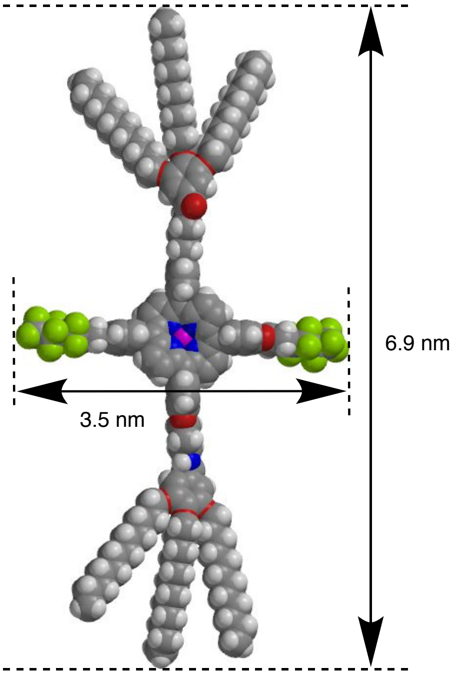

Supplementary Figure 11
Computer-generated molecular model of **6FF**. Atom color code: pink, Zn; grey, C; red, O; blue, N; green, F; white, H.

ADF–STEM measurements

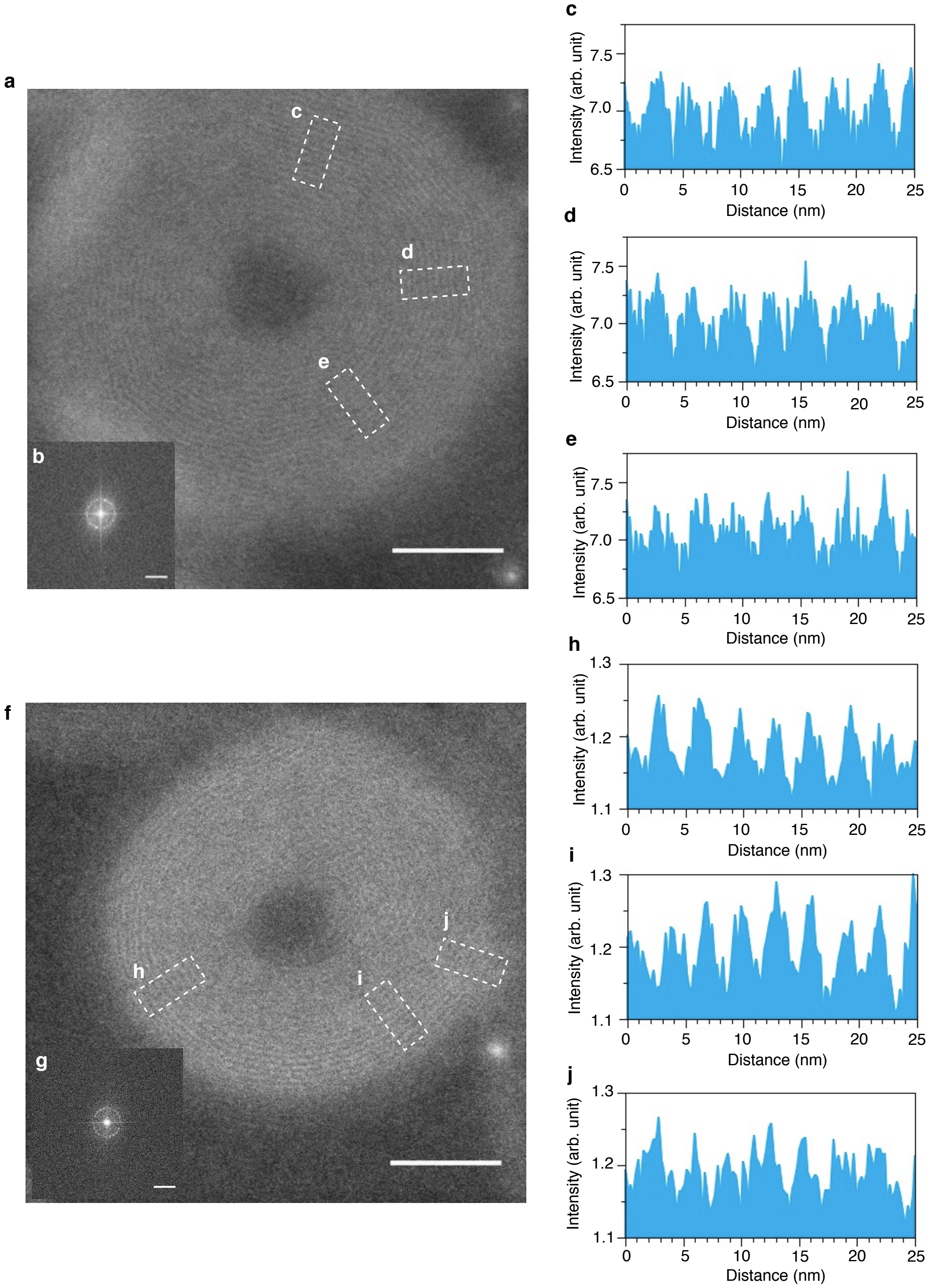

Supplementary Figure 12

(a,f) ADF–STEM images of Archimedean spiral of **6FF**. Scale bar = 50 nm. (b,g) Fast Fourier transform of the image, revealing a periodicity of ca. 3.1 nm, which corresponds to the average separation distance between consecutive turns. Scale bar = 0.5 nm^{-1}. (c-e, h-j) Cross-sectional histogram; an average periodicity of ca. 3.2 nm was obtained from the difference in electron density.

SAXS measurement

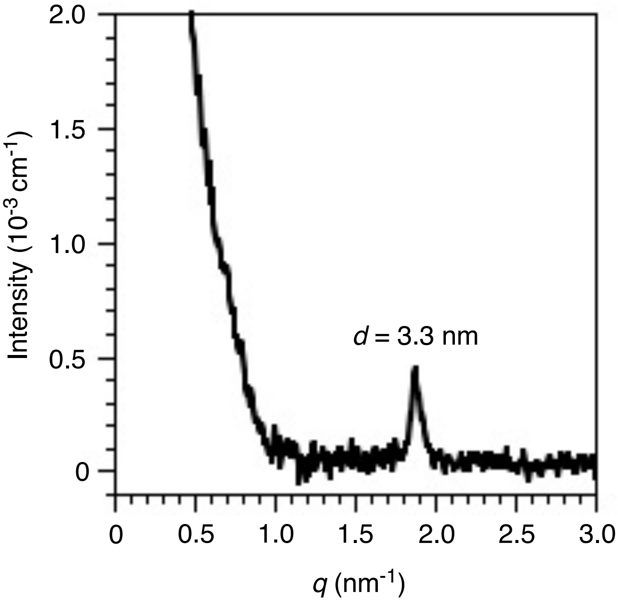

Supplementary Figure 13
SAXS profile of Archimedean spirals of **6FF** (20 μM) in dodecane at 298 K.

AFM images of defects in Archimedean spirals of 6FF

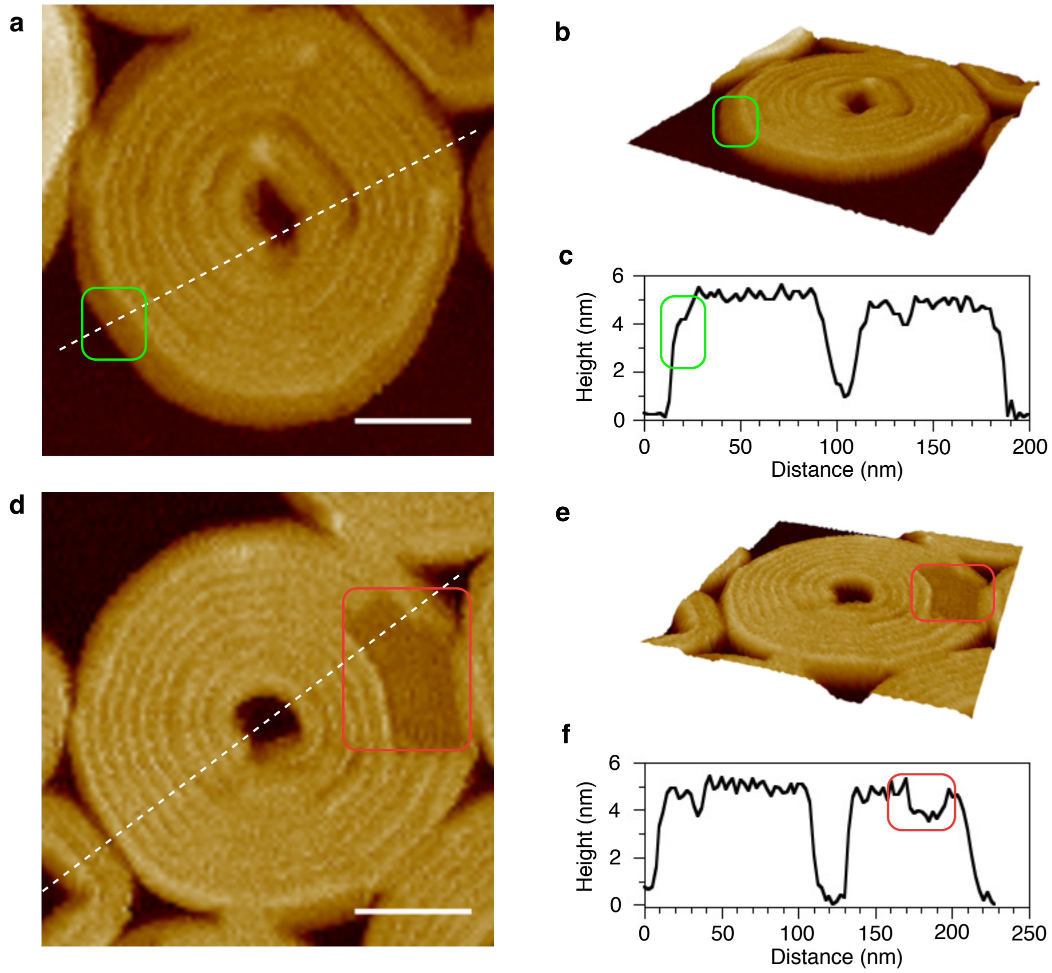

Supplementary Figure 14
(a,d) AFM images, (b,e) 3D AFM images, and (c,f) height profiles of Archimedean spirals of **6FF** obtained from a dodecane solution (12.5 μM): scale bar = 50 nm.

Schematic representation of molecular packing of 6FF in Archimedean spiral

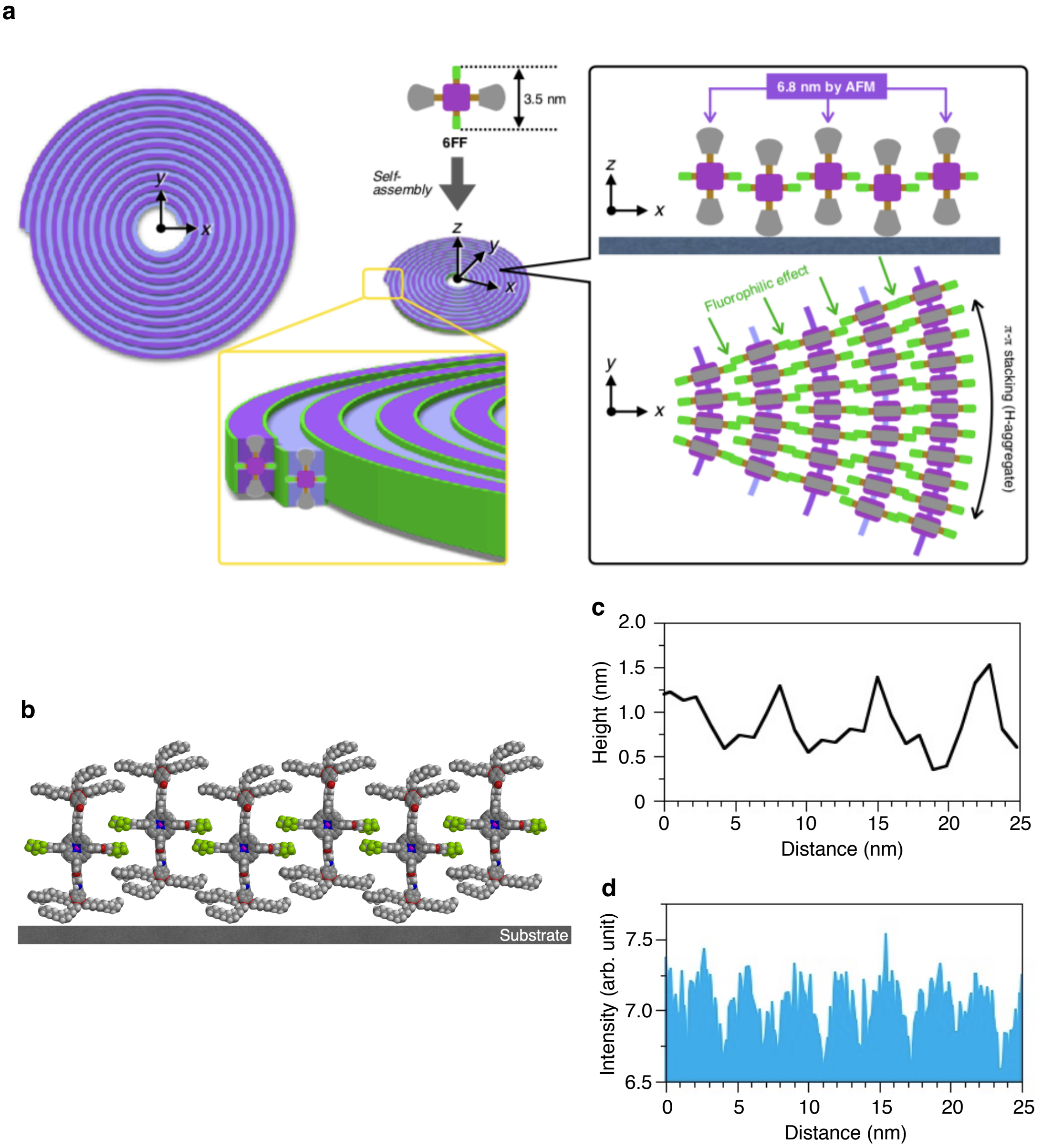

Supplementary Figure 15

(a) Schematic representation of self-assembly of **6FF** into Archimedean spiral. (b) Proposed packing structure of **6FF** in Archimedean spiral. (c) Height profile of Archimedean spirals of **6FF** obtained from an AFM image. (d) Cross-sectional histogram calculated from the difference in electron density in a TEM image.

FT-IR spectrum of Archimedean spiral of 6FF

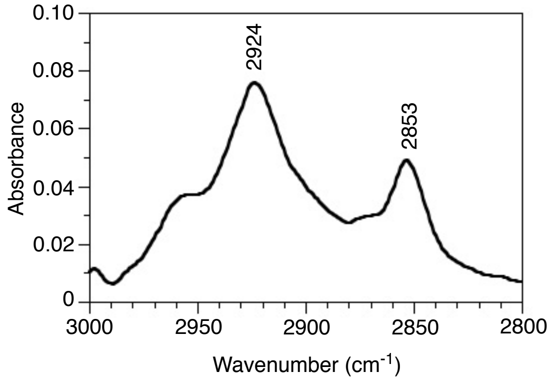

Supplementary Figure 16
FT-IR spectrum of Archimedean spirals of **6FF**. The peaks at 2924 and 2853 cm^{-1} suggest that the dodecyl chains in **6FF** adopt a folded gauche conformation.[6]

AFM images of Archimedean spirals of 6FF

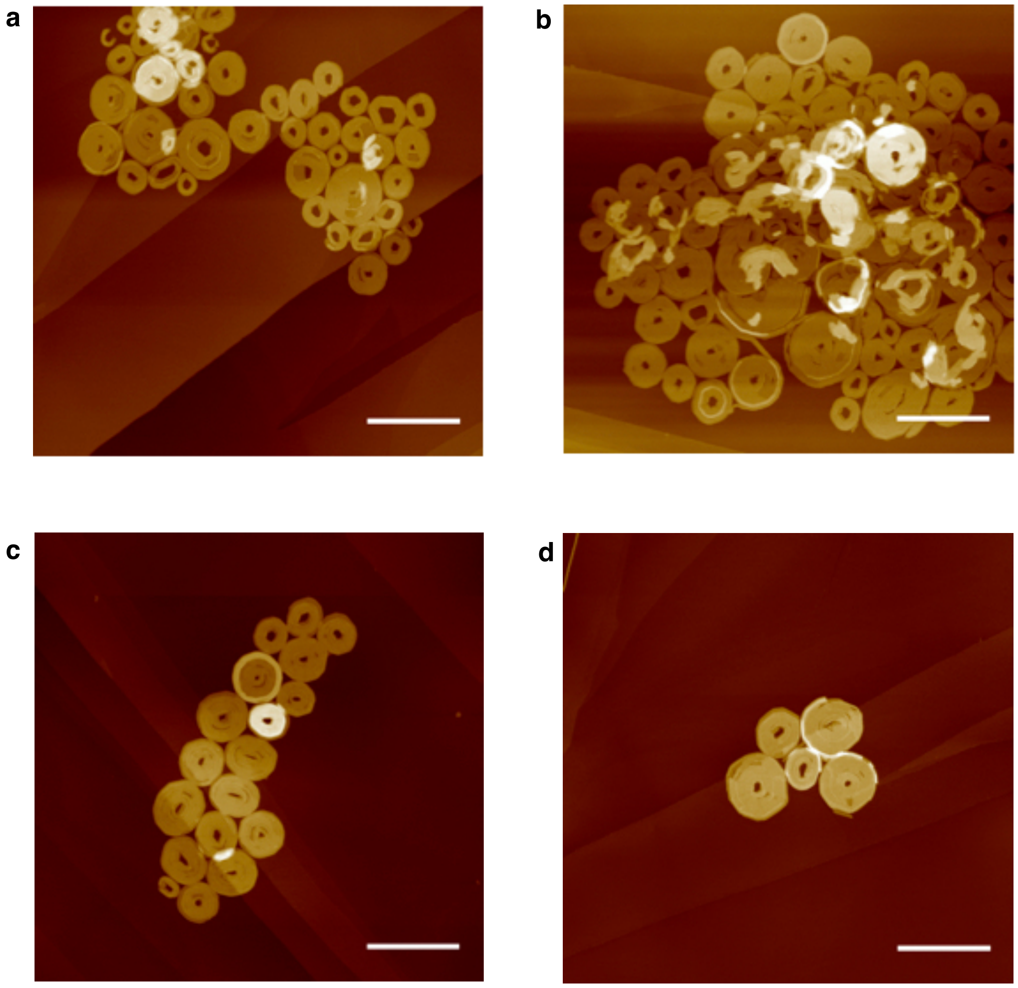

Supplementary Figure 17
AFM images of Archimedean spirals of **6FF** obtained from a hot dodecane solution (12.5 μM) upon cooling at rates of (a) −10 K/min, (b) −5 K/min, (c) −3.4 K/min, and (d) −1.7 K/min on HOPG substrate: scale bar = 400 nm.

Size distribution of Archimedean spiral

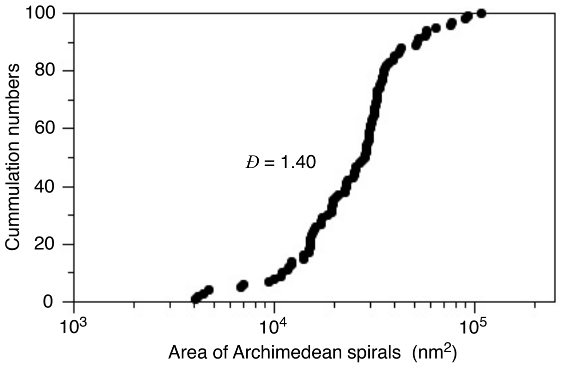

Supplementary Figure 18
Cumulative histogram of the area of Archimedean spirals of **6FF** obtained by "quenching" a hot dodecane solution (12.5 μM) (Fig. 3g). The data were collected by tracing 100 objects in AFM images.

Absorption spectra of porphyrin derivatives in different forms

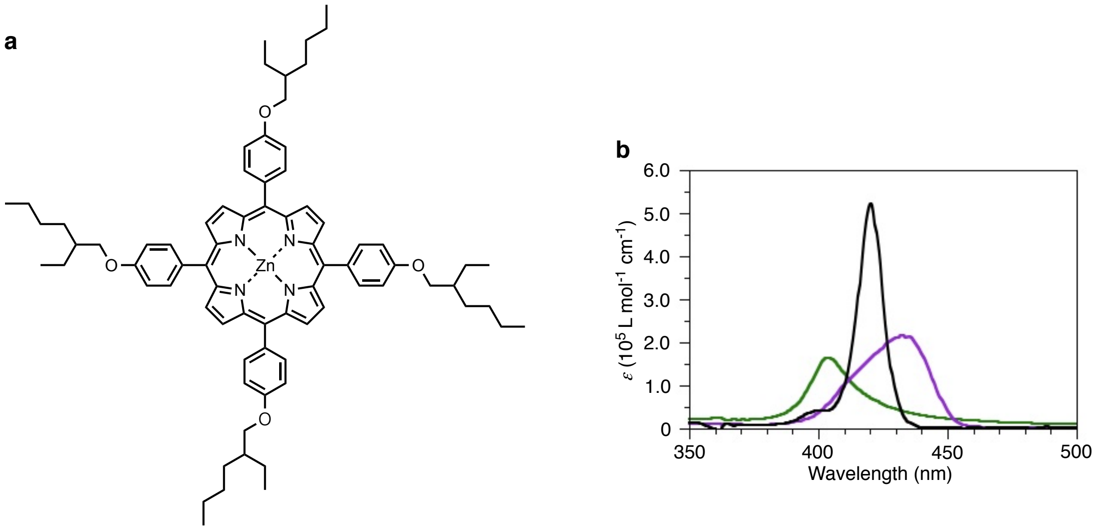

1R

Supplementary Figure 19
(a) Structure of non-assembling porphyrin (**1R**). (b) Absorption spectra of non-assembling porphyrin (**1R**) in a monomeric form (black) in dodecane: [**1R**] = 12.5 μM at 298 K, J-aggregates of **6FF** (purple) in dodecane: [**6FF**] = 50 μM at 283 K, and H-aggregated Archimedean spirals of **6FF** (green) in dodecane: [**6FF**] = 12.5 μM at 303 K.

Precipitation of Archimedean spiral of 6FF in dodecane

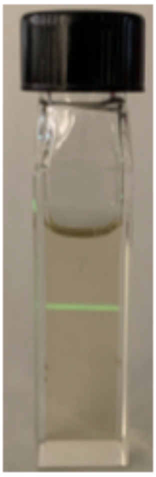

Supplementary Figure 20
Photograph of precipitated Archimedean spirals of **6FF** in dodecane under irradiation with a green laser pointer, showing Tyndall effect: [**6FF**] = 12.5 μM at 298 K.

Details on the thermodynamic mass balance model and fits

The supramolecular polymerization of the monomers is modelled as a sequence of monomer additions to the chain ends of growing polymers. In the model, a nucleated cooperative supramolecular polymer is in equilibrium with the free monomers and a competing isodesmic polymerization pathway. Assuming a nucleus size of 2, the mass balance equation for this system is:

$$[\mathrm{M}]_{\mathrm{tot}} = -\sigma \cdot [\mathrm{M}] + \frac{\sigma \cdot [\mathrm{M}]}{(1-K_{\mathrm{e}} \cdot [\mathrm{M}])^2} + \frac{[\mathrm{M}]}{(1-K_{\mathrm{iso}} \cdot [\mathrm{M}])^2} \quad \text{(S1)}$$

with $[\mathrm{M}]_{\mathrm{tot}}$ the total amount of material present in the system, σ the cooperativity parameter, [M] the free monomer concentration and K_{e} and K_{iso} the associations constants of monomer addition to the growing nucleated and isodesmic polymer respectively. The cooperativity parameter, σ, is defined as $K_{\mathrm{n}}/K_{\mathrm{e}}$, with K_{n} the equilibrium constant for nucleation of the cooperative polymer. A full derivation of the mass balance equation can be found elsewhere.[7] The critical aggregation concentration at 298 K of the nucleated supramolecular polymers was obtained by calculating the maximum free monomer concentration at that temperature, which is equal to the inverse of the elongation constant of the polymerization.

The temperature dependency of the equilibrium constants is introduced via the van ‘t Hoff equation:

$$K_i = \exp\left(\frac{-\Delta G_i}{R \cdot T}\right) = \exp\left(\frac{-\Delta H_i}{R \cdot T} + \frac{\Delta S_i}{R}\right) \quad \text{(S2)}$$

with ΔG_i the Gibbs free energy of each process i, that has an enthalpy and entropy contribution of ΔH_i and ΔS_i respectively and R the gas constant. Furthermore, the nucleation enthalpy is related to the elongation enthalpy via:

$$\Delta H_{\mathrm{n}} = \Delta H_{\mathrm{e}} + NP \quad \text{(S3)}$$

with NP the nucleation penalty. The entropies of the nucleation and elongation phases are assumed to be equal.

The model is fitted the absorption signals at 403 and 434 nm simultaneously for multiple concentrations in a global fit. The absorption spectra of the H-aggregated Archimedean spirals of **6FF** and **6FH** are obtained at 303 K and 293 K respectively from a cooled solution at a rate of 0.9 K min^{-1} of either monomer, while the absorption spectra of the J-aggregate of **6FF** and **6FH** are obtained at respectively 283 K from a rapidly cooled, “quenched”, solution. The absorption coefficient of the monomer is obtained from the spectrum of a non-assembling reference porphyrin (**1R**). The values for these coefficients are given in Supplementary Table 2. All absorption coefficients were corrected in the fit for a temperature dependent drift due to expansion of the solvent (Supplementary Figure 21). This temperature dependent drift of 0.1545% K^{-1} was used to calculate the relative change of the absorption coefficients upon changing temperature. Calculated VT-UV-traces at 403 and 434 nm were constructed by calculating the concentration of all species in solution using equations (S1) and (S2), after which these concentrations were multiplied by the absorption coefficients and the path length (10 mm) to obtain a calculated temperature dependent VT-UV traces at 403 and 434 nm.

Supplementary Table 2
Absorption coefficients of the J-aggregate and H-aggregate in M^{-1} cm^{-1}.

	6FF		6FH	
	403 nm	434 nm	403 nm	434 nm
**J-aggregate**	51600[a]	212000[a]	45600[a]	234000[a]
**H-aggregate**	164000[b]	38600[b]	177000[c]	50900[c]

[a]Values obtained at 283 K; [b]Values obtained at 303 K; [c]Values obtained at 293 K.

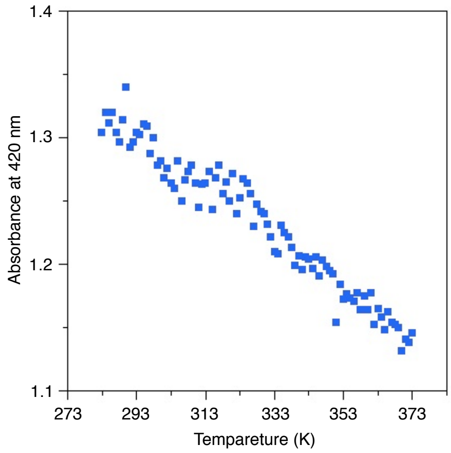

Supplementary Figure 21
Temperature dependent absorbance at 420 nm of **1R** (12.5 μM), showing the decrease in absorption upon elevating the temperature due to expansion of the dodecane solvent.

The calculated VT-UV traces at 403 and 434 nm were fitted in a global fitting routine, where all data is fitted simultaneously, to the experimental data using least square optimization minimization. In this optimization, a cost vector, which is obtained by subtracting the predicted UV trace from the experimentally obtained data, is minimized. To ensure realistic solutions, where the H-aggregates are favored over the J-aggregates at low temperatures, as well as efficient fitting, cost vectors for thermodynamic parameter sets with a more negative entropy of isodesmic aggregation than the entropy of elongation, $\Delta S_{iso} < \Delta S_e$, were multiplied by 1500, effectively rendering them very poor solutions. The fit was performed on 250 initial parameter sets that were sampled using Latin hypercube sampling. In the sampling, Gibbs free energies of the elongation phase of the cooperative polymerization and of the isodesmic polymerization were sampled between −33 and −50 kJ mol^{-1} and −25 and −53 kJ mol^{-1}, respectively. Nucleation penalties were sampled between 5 and 30 kJ/mole and entropies of the cooperative and isodesmic polymer were sampled between −30 and −300 J mol^{-1} K^{-1}. The randomly sampled Gibbs free energies and corresponding entropies were used to generate reasonable random starting values for the enthalpies of the elongation of the cooperative polymer and isodesmic aggregation. The best fit was obtained by selecting the optimized parameter set that resulted in the lowest norm of the residual cost vector. In all reported fits, a unique dataset that described the experimental data was found.

Correlations in fit results of 6FF

In our initial attempts to fit the data of **6FF**, global fits on various concentrations ranging from 2.5 to 15 μM were performed, but these fits did not yield a unique set of thermodynamic parameters that properly describes the data. When only concentrations of 10 μM or higher are taken into account, which is the concentration regime where only the Archimedean spirals and not the concentric toroids are present, the fits gave a unique solution. These results thus indicate that the formation of the Archimedean spirals can be accurately described by the above described mass-balance model involving an isodesmic and cooperative pathway. Moreover, the thermodynamic properties of the concentric toroids must be subtly different. As a result, the model cannot accurately describe the data and no unique set of fit parameters can be found when concentrations where the concentric toroids are formed are used in the fit.

This behavior is illustrated by looking at the correlations between the optimized fit parameters of all optimized fits that are within 5% of the best fit for the various fits performed. In these plots, a matrix of scatter plots is presented which shows the correlation between every pair of fit parameters (ΔH_e, ΔH_{iso}, NP, ΔS_e and ΔS_{iso}). When (linear) trends can be observed, there is no unique solution, as the algorithm can achieve equally good fits by concomittantly changing those two thermodynamic parameters. Thus, only when the results are scattered in a narrow regime, a single solution is found. This narrow range over which the optimized parameters are scattered is obtained when only concentrations of 10 μM or higher are taken into consideration in the fit.

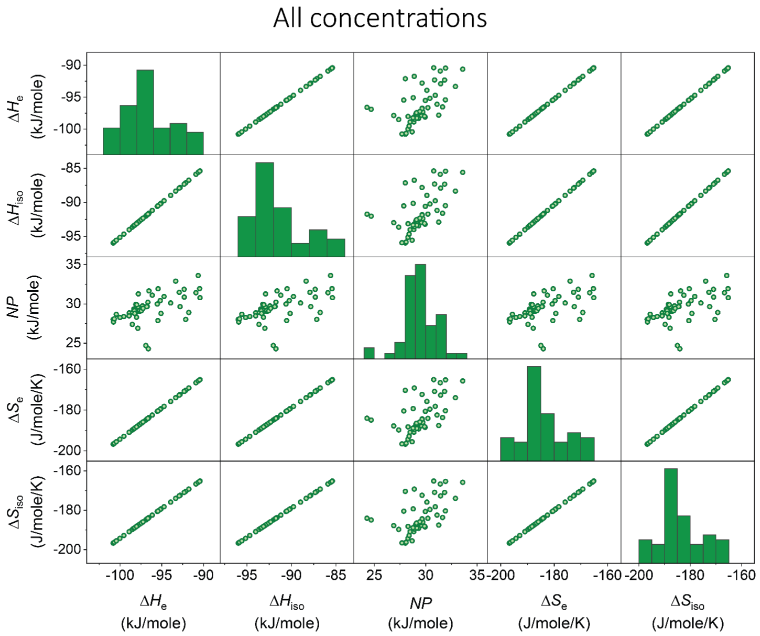

Supplementary Figure 22

Matrix of scatter plots showing the optimized parameter sets with a residual < 5% from the fit with lowest residual when data of 2.5, 5.0, 7.5, 10.0, 12.5 and 15.0 μM of **6FF** are fitted. The linear trends indicate that the optimized parameters are correlated and *the thermodynamic parameters cannot be uniquely determined.*

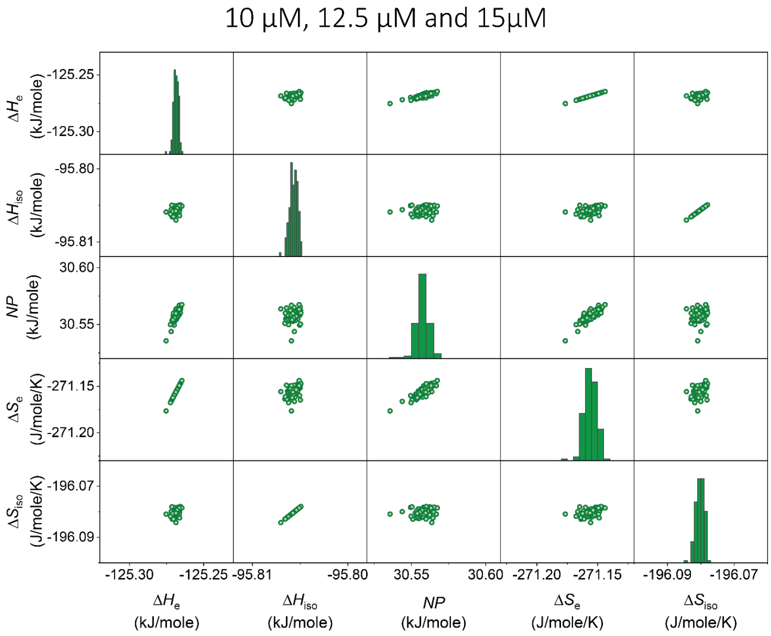

Supplementary Figure 23

Matrix of scatter plots showing the optimized parameter sets with a residual < 5% from the fit with lowest residual when data of 10.0, 12.5 and 15.0 µM of **6FF** are fitted. The spread of the values over a small regime indicates that a unique solution is found and *the thermodynamic parameters are accurately obtained* (Supplementary Table 3).

Supplementary Table 3

Optimized fit parameters obtained from the best fit for concentrations of **6FF** of 10, 12.5 and 15 µM

	ΔH_e<br>kJ mol^{-1}	ΔH_{iso}<br>kJ mol^{-1}	NP<br>kJ mol^{-1}	ΔS_e<br>J mol^{-1} K^{-1}	ΔS_{iso}<br>J mol^{-1}K^{-1}
Optimized values	−125	−95.8	30.6	−271	−196

Archimedean spirals of 6FH

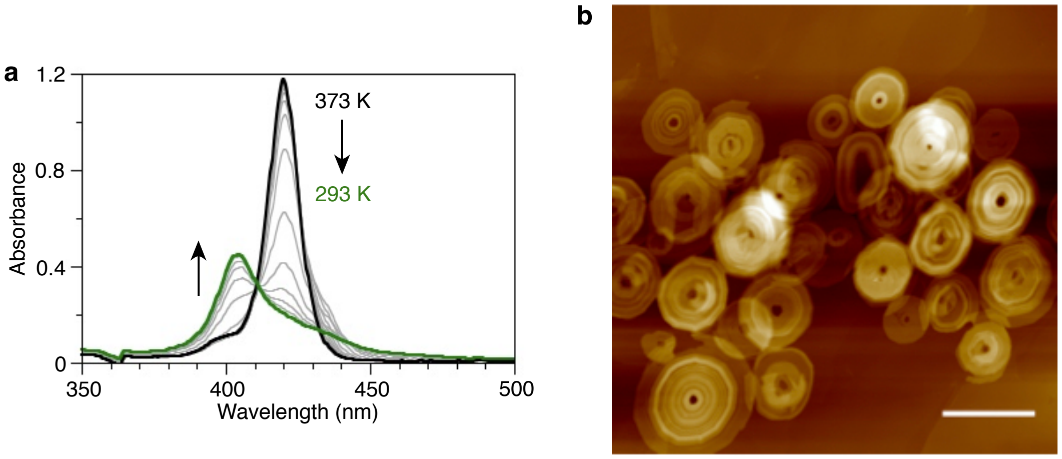

Supplementary Figure 24
(a) Temperature-dependent changes in the absorption spectrum of a solution of **6FH** in dodecane: [**6FH**] = 12.5 µM, −0.5 K/min. (b) AFM image of Archimedean spirals of **6FH** on HOPG substrate: scale bar = 600 nm

Correlations in fit results of 6FH

Similar to the results obtained for **6FF**, the fits of **6FH** that incorporated the lower concentrations, did not yield a unique solution, while the fits that incorporated only the concentrations of 12.5 μM and higher did give a unique solution, indicated by the spread of the fit parameters of optimized fits within 5% of the best fit over a small range.

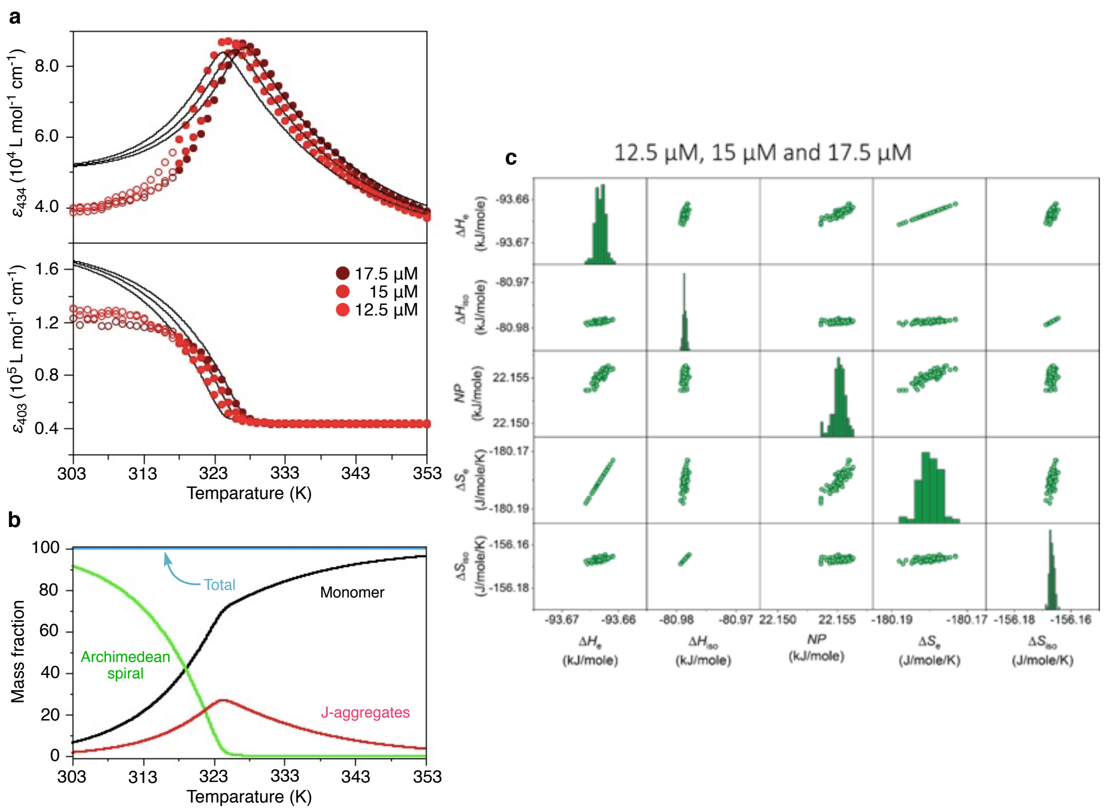

Supplementary Figure 25

(a) Plots of molar absorption coefficients at 434 and 403 nm as a function of temperature: [**6FH**] = 12.5, 15.0, 17.5 μM; −0.5 K/min. The two-pathway thermodynamic model was fitted (solid lines) to the plots indicated by the reddish filled marks. (b) Calculated temperature-dependent speciation plot of a 12.5 μM solution of **6FH**. The speciation plot shows the distribution of monomers over the free monomeric, J-aggregated, and H-aggregated (Archimedean spiral) states. (c) Matrix of scatter plots showing the optimized parameter sets with a residual <5% from the fit with lowest residual when data of 12.5, 15.0 and 17.5 μM of **6FH** are fitted. The spread of the values over a small regime indicates that a unique solution is found and *the thermodynamic parameters are accurately obtained* (Supplementary Table 4).

Supplementary Table 4
Optimized fit parameters obtained from the best fit for concentrations of **6FH** of 12.5, 15, and 17.5 µM

	ΔH_e kJ mol^{-1}	ΔH_{iso} kJ mol^{-1}	NP kJ mol^{-1}	ΔS_e J mol^{-1} K^{-1}	ΔS_{iso} J mol^{-1}K^{-1}
Optimized values	−93.7	−81.0	22.1	−180	−156

Calculated speciation plots

From the fits of the experimental data to the mass balance model, the distribution of the monomers over the isodesmic and cooperative can be calculated[7]. The results hereof are given in Figure 4c and Supplementary Figure 26.

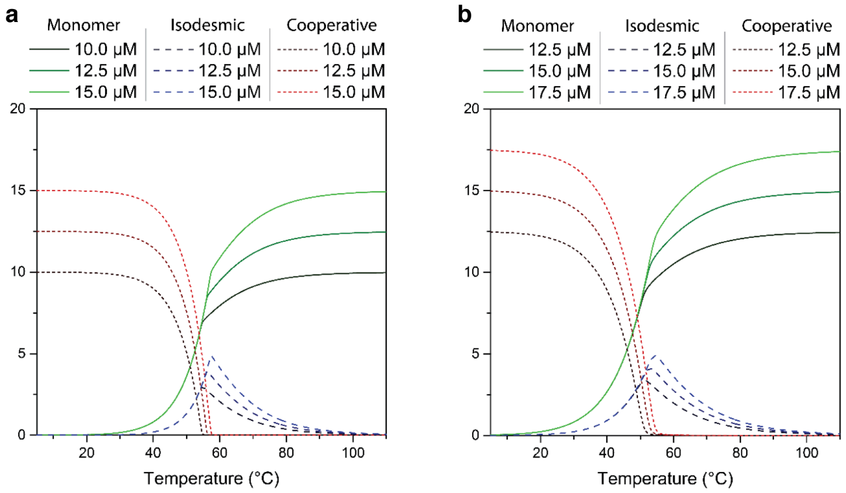

Supplementary Figure 26
Calculated speciation plots, showing the distribution of monomers over the isodesmic J-aggregates and cooperative H-aggregates for (a) **6FF** and b) **6FH**.

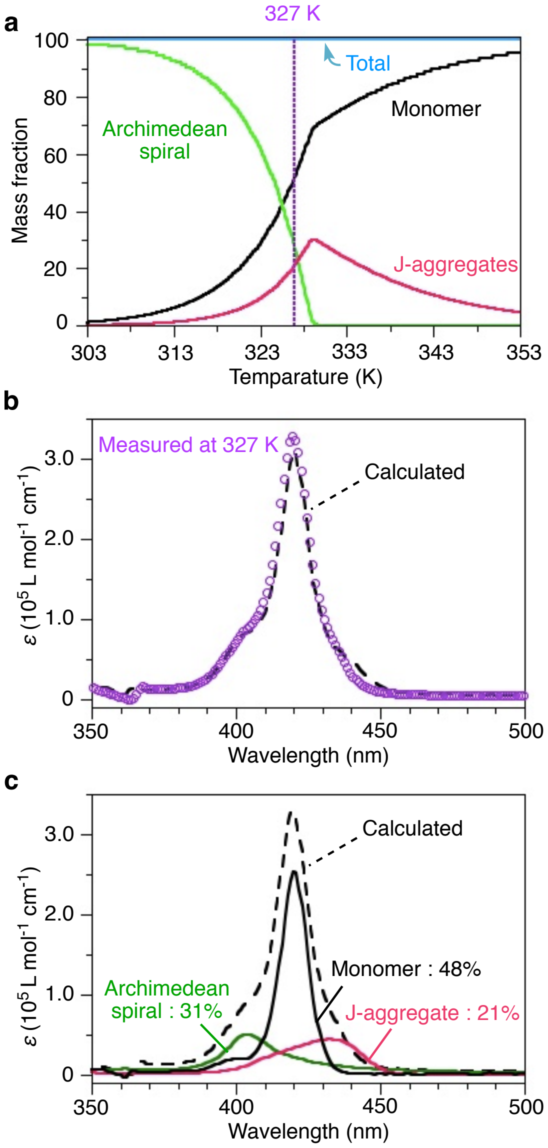

Supplementary Figure 27

(a) Calculated temperature-dependent speciation plot of a 12.5 μM solution of **6FF**. The speciation plot shows the distribution of monomers over the free monomeric, J-aggregated, and H-aggregated (Archimedean spiral) states. At 327 K, the distribution of monomers over free monomeric, J-aggregated, and H-aggregated (Archimedean spiral) states are 48, 21, and 31%, respectively. (b) Comparison of experimentally obtained spectrum at 327 K (purple open circle) and calculated absorption spectrum (black dashed line). (c) Calculated absorption spectrum (black dashed line) on the basis of the distribution of monomers at 327 K over the free monomeric (black line), J-aggregated (pink line), and H-aggregated (Archimedean spiral: green line) states.

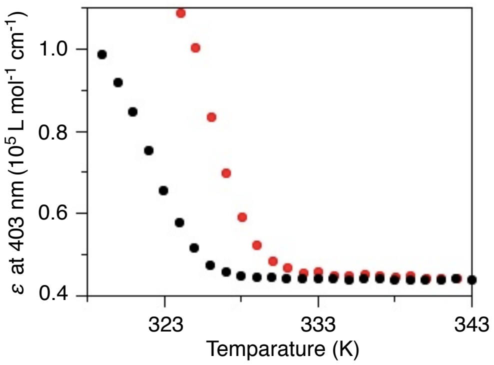

Supplementary Figure 28
Plots of molar absorption coefficients of **6FF** (red circle) and **6FH** (black circle) in dodecane at 403 nm as a function of temperature: [**6FF**] = [**6FH**] = 12.5 μM; −0.5 K/min.

ADF–STEM measurements

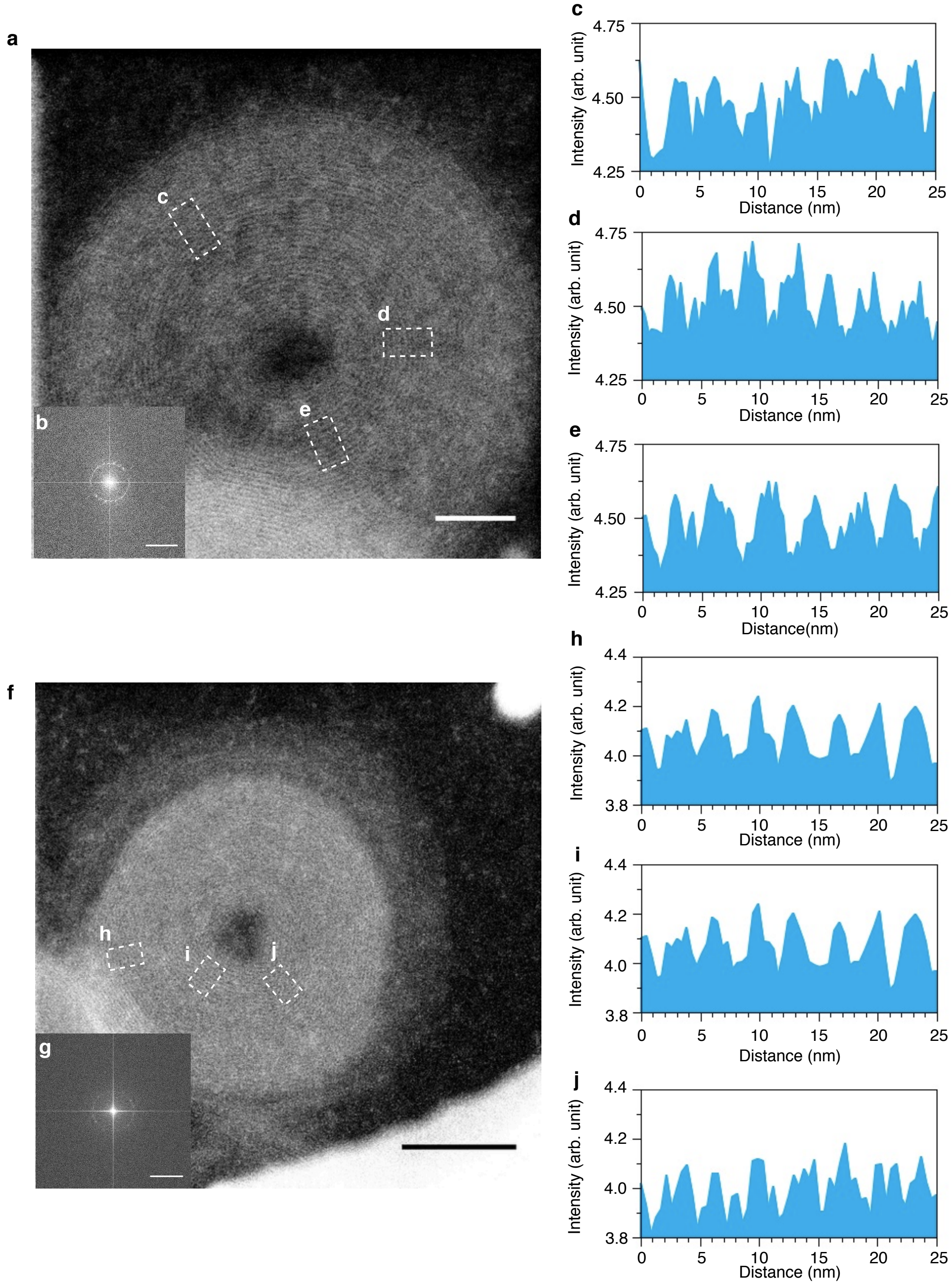

Supplementary Figure 29
(a,f) ADF–STEM images of concentric toroid of **6FF**. Scale bar = 50 nm (a) and 100 nm (b). (b,g) Fast Fourier transform of the image, revealing a periodicity of 3.1 nm, which corresponds to the average separation distance between consecutive turns. Scale bar = 0.5 nm^{-1}. (c-e, h-j) Cross-sectional histogram; an average periodicity of ca. 3.2 nm was obtained from the difference in electron density.

Absorption spectra of Archimedean spirals and concentric toroids of 6FF

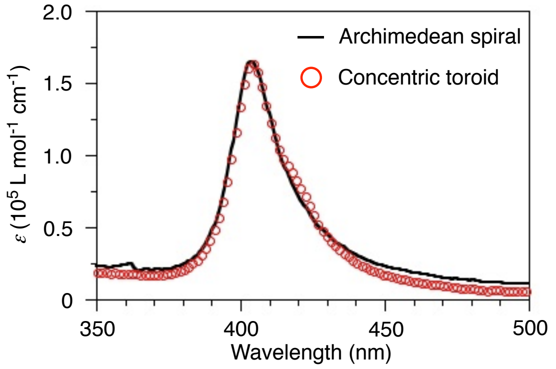

Supplementary Figure 30
Absorption spectra of Archimedean spirals and concentric toroids of **6FF** in dodecane: [**6FF**] = 12.5 µM in Archimedean spirals, [**6FF**] = 7.5 µM in concentric toroids, at 303 K.

Correlations in fit results of 6FF

2.5 µM, 5.0 µM and 7.5µM

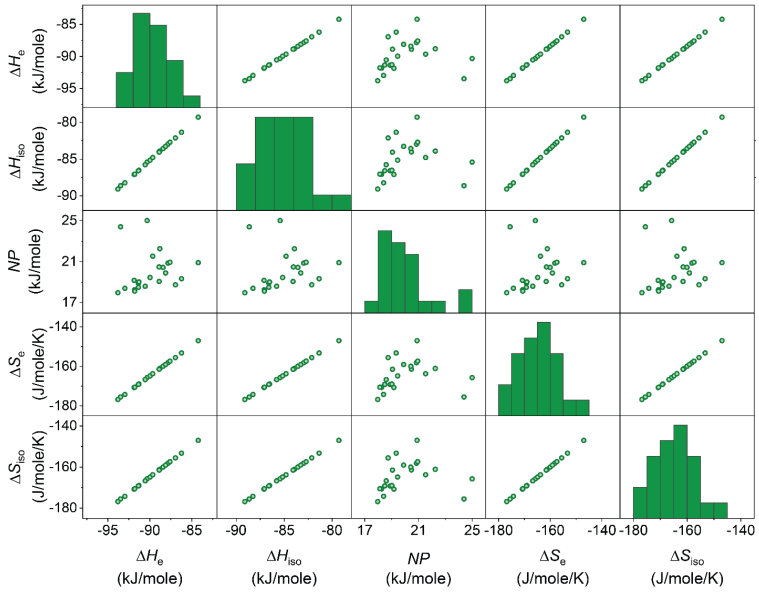

Supplementary Figure 31
Matrix of scatter plots showing the optimized parameter sets with a residual < 5% from the fit with lowest residual when concentrations of **6FF** between 2.5 and 7.5 µM are fitted. The linear trends indicate that the optimized parameters are correlated and *the thermodynamic parameters cannot be uniquely determined.*

AFM images of self-assembled structures of 6FH

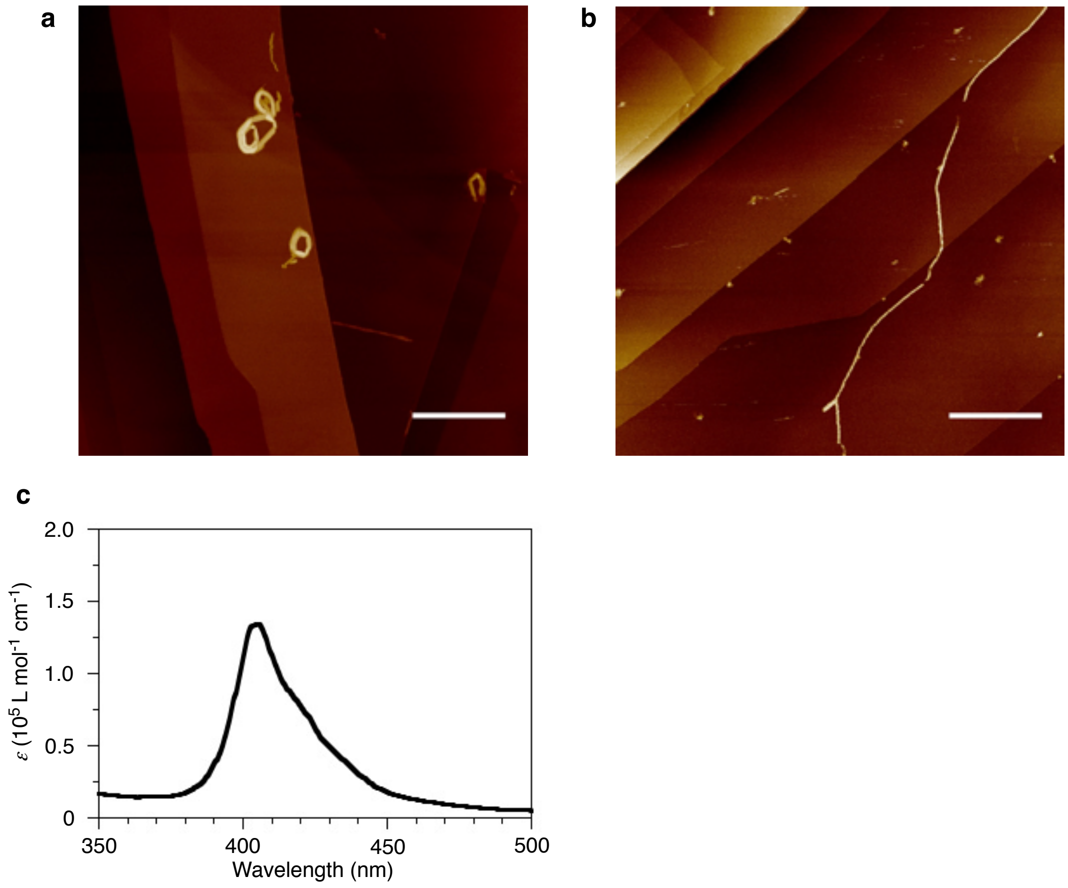

Supplementary Figure 32
(a,b) AFM images: HOPG substrate, scale bar = 400 nm, and (c) absorption spectrum of H-aggregates of **6FH** obtained by cooling a hot dodecane solution (10 µM).

Size distribution of concentric toroids

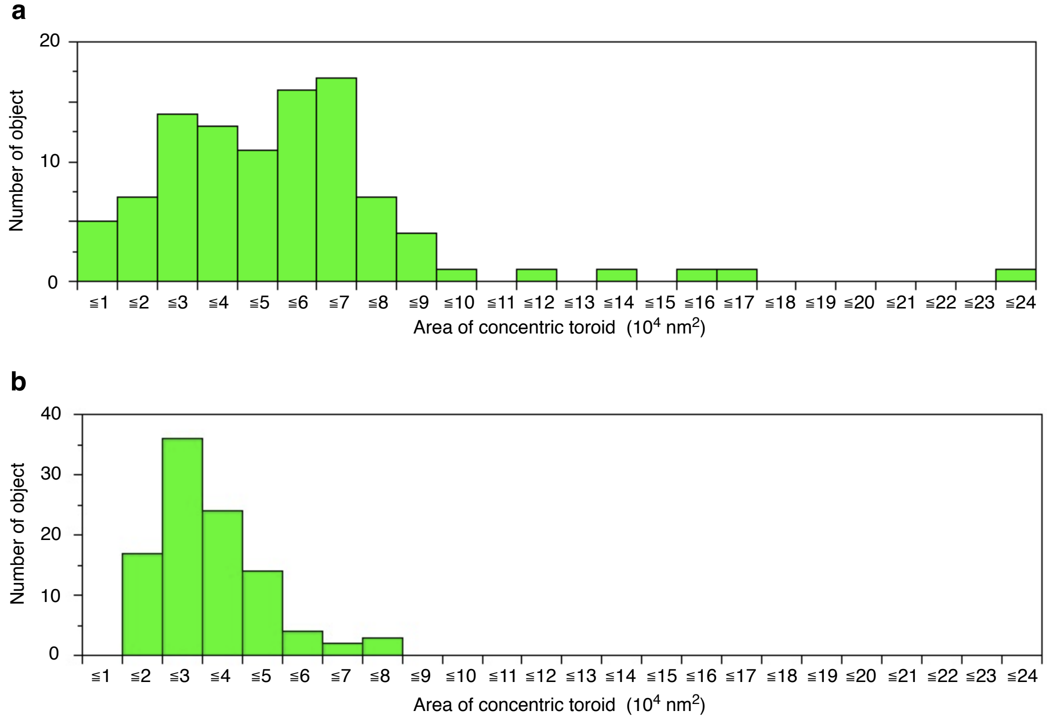

Supplementary Figure 33

Histograms of the area of concentric toroid of **6FF** obtained by (a) cooling a hot dodecane solution (7.5 μM) and (b) the solvent mixing protocol (5.0 μM in toluene–dodecane = 1:19 (v/v)). The data were collected by tracing 100 objects in AFM images.

4. Supplementary References

1. Fukui, T., Kawai, S., Fujinuma, S., Matsushita, Y., Yasuda, T., Sakurai, T., Seki, S., Takeuchi, M. & Sugiyasu, K. Control over differentiation of a metastable supramolecular assembly in one and two dimensions. *Nature. Chem.* **9**, 493-499 (2017).
2. Ogi, S., Sugiyasu, K., Manna, S., Samitsu, S. & Takeuchi, M. Living supramolecular polymerization realized through a biomimetic approach. *Nature Chem.* **6**, 188-195 (2014).
3. Neubauer, T., Kammerer-Pentier, C. & Bach, T. Total synthesis of (+)-bretonin B: access to the (*E*, *Z*, *E*)-triene core by a late-stage Peterson elimination of a convergently assembled silyl ether. *Chem. Commum* **48**, 11629-11631 (2012).
4. Takagi, H., Igarashi, N., Nagatani, Y., Ohta, H., Mori, T., Kosuge, T. & Shimizu, N. New high-brilliance small angle x-ray scattering beamline, BL-15A2 at the photon factory. *AIP Conf. Proc.* **2054**, 060038 (2019).
5. Shimizu, N., Yatabe, K., Nagatani, Y., Saijyo, S., Kosuge, T. & Igarashi, N. Software development for analysis of small-angle x-ray scattering data. *AIP Conf. Proc.* **1741**, 050017 (2016).
6. Snyder, R. G. & Strauss, H. L. C-H stretching modes and structure of n-alkyl chains. 1. Long, Disordered chains. *J. Phys. Chem.* **86**, 5145-5150 (1982).
7. Mabesoone, M. F. J., Markvoort, A. J., Banno. M., Yamaguchi, T., Helmich. F., Naito. Y., Yashima. E., Palmans. A. R. A. & Meijer. E. W. Competing interactions in hierarchical porphyrin self-assembly introduce robustness in pathway complexity. *J. Am. Chem. Soc.* **140**, 7810-7819 (2018).
